# Morphodynamics of non-canonical autophagic structures in *Neurospora crassa*

**DOI:** 10.1128/msphere.00460-23

**Published:** 2023-10-17

**Authors:** Alberto Rivetta, Kenneth Allen, Morven Graham, Tatiana Potapova, Clifford Slayman, Xinran Liu

**Affiliations:** 1 Department of Cellular and Molecular Physiology, Yale School of Medicine, New Haven, Connecticut, USA; 2 Department of Cell Biology, Yale School of Medicine, New Haven, Connecticut, USA; 3 Belozersky Institute of Physicochemical Biology, Moscow State University, Moscow, Russia; University of Georgia, Athens, Georgia, USA

**Keywords:** vacuole proliferation, phagophore branching, doubled phagophores, autophagosomes, electron tomography, plasma membrane in-growth, lysosomes, microvesicles, mycelial fungi

## Abstract

Autophagy is a major pathway for unspooling cytoplasmic constituents and recycling them. Here, we investigate autophagy in *Neurospora crassa,* a close ascomycete relative to the model yeast *Saccharomyces cerevisiae*. High-pressure-freeze and freeze-substitution techniques proved keys to preservation of the surprising autophagic structures observed in *Neurospora* by transmission electron microscopy and electron tomography. Depriving *Neurospora* of carbon triggers two parallel processes at the plasma membrane: formation of vacuoles, initially along the plasma membrane but stuffing 70%–80% of cytoplasmic volume after several hours. The vacuoles contain material left over from digested proteins, disrupted ribosomes or glycogen clumps, plus other discrete organelle-like objects. Also, they accumulate the canonical autophagy marker Atg8, appear to be lytic from the start, and probably grow from lysosomes. Formation of diverse phagophores and autophagosomes, *multi-membrane* organelles manufactured *de novo* by the plasma membrane—a feature so far unique to *Neurospora*. These autophagic structures can be observed attached either to the cell membrane, to the cell wall, or free in the cytoplasm, viz. as mature organelles after detachment from the cell membrane. Although phagophores are clearly observed in carbon-replete cells, their production increases with carbon starvation—and these, too, often appear lytic. *ATG1*, the canonical initiator of phagophore formation in yeast, is not required for the formation of phagophores in *Neurospora*. This work provides clear structural and functional evidence that *Neurospora*’s autophagic organelles differ from those observed by others in yeast.

**IMPORTANCE:**

*Neurospora* is a quintessential tip-growing organism, which is well known for packaging and longitudinal transport of tip-building blocks. Thus far, however, little attention has been paid to the co-essential process of reclamation, that is—taking apart of upstream, older structural elements, otherwise known as “autophagy”. We are not yet prepared to set out the chemistry of that elaborate process, but its morphological start alone is worthy of attention. Carbon starvation triggers significant autophagic changes, beginning with prolific vacuolation along the plasma membrane, and eventual filling of 70% (or more) of cytoplasmic volume. Additionally, the *Neurospora* plasma membrane elaborates a variety of phagophores which themselves often look lytic. These have either dual enclosing membranes, like the familiar autophagosomes, can be doubled and have four wrapping membranes, or can be compounded with multiple membrane layers. These reclamation processes must be accommodated by the mechanism of tip growth.

## INTRODUCTION

It is now understood that cyclic degradation, recovery, and reusing of molecular constituents is fundamental to the maintenance of living cells, that is—to life itself. Multiple studies have shown that serious disruption of recycling pathways shortens lifetime and diverts or weakens particular functions in complex organisms. Although the biochemical pathways involved in these degradation and recycling processes differ considerably from species to species, and are still being worked out, the major pathways in eukaryotes are mediated by the ubiquitin-proteasome system and the vacuolar-lysosomal-autophagy mechanism.

It has been known for more than 80 years that the removal of common nutrients from the environment of free-living microorganisms leads to the upregulated production of scavenging systems—high-affinity uptake systems—for the depleted nutrients. This was elegantly demonstrated in the ascomycete *Neurospora* by Schneider and Wiley, in 1971, for the monosaccharides glucose, fructose, galactose, and lactose (1), and later for those and other sugars in *Saccharomyces* (2–4). It had also been discovered that certain nutrient depletions provoked morphological changes: proliferation of vesicles (lysosomes) and formation of frank vacuoles associated with the recycling of molecular components of cytoplasm, as first observed by de Duve, in 1963, and dubbed autophagy (5, 6). By the early 1990s autophagic recycling had become a major area of investigation, pursued especially vigorously in *Saccharomyces* (7–11), in multiple animal-cell preparations (12–14), in plants (15–18), and in mycelial fungi as well (19–21).

Working on *Neurospora* for other reasons—electrophysiological study of membrane transport—we noticed that carbon starvation as carried out by Schneider and Wiley (1, 22) also produced numerous small vacuoles (23). These were especially prominent during the removal of the carbon/energy source, but the removal of nitrogen or phosphate gave qualitatively similar results (Slayman and Potapova, unpub). The same maneuvers also yielded *hyper*polarization of the plasma membrane, from approx. −180 to −220 mV, under standard lab conditions. The voltage change was accompanied by a 7–10-fold *increase* in electrical resistance of the plasma membrane (23), saving energy by downregulating ion transport systems. The modest hyperpolarization implies a weaker downregulating effect on the plasma membrane’s proton pump (*Pma1*) than upon the ensemble membrane resistance. Thus, selective *up*regulation of high-affinity sugar transport (1) was just a direct expression of the scavenging function.

A significant special observation with *Neurospora* has been that the morphological changes, but not the electrical changes, occur upon removal of glucose alone, while carbon/energy is supplied by other sugars (e.g., fructose or galactose), so implying that vacuolation is related *preferentially to glucose signaling*, not solely to carbon/energy starvation. However, the detailed biochemical processes which underlie these morphological and physiological changes remain largely to be characterized. That subject is now interesting because of recent spectacular progress in autophagy research on other systems: especially on *Saccharomyces* (7–11) and cultured mammalian cells (12–14). Starvation for phosphate, nitrogen, or specific amino acids (in auxotrophs) also triggers vacuolation in *Neurospora* as noted above, but we have pursued glucose starvation, because it is most conspicuous in hyphae of any diameter, and very reproducible. A direct comparison between carbon-starvation and nitrogen-starvation is shown in Fig. S1.

The purpose of this manuscript is, therefore, to set down a clear temporal description of starvation-induced *morphological* transformations, which are easily seen in the older (upstream) segments of hyphae, but not so easily in the younger segments, just behind the growing tips. The characteristic growth of *Neurospora* (and many species of mycelial fungi) is supported by longitudinal streaming, due both to pressure-driven bulk flow and to kinesin-powered sliding or walking of vesicles along microtubules, toward a Vesicle-Supply Center (i.e., spitzenkörper) just behind the tip. This means that autophagy in the older segments should be a ready and direct source of recyclable molecules for growth at hyphal tips. Historically, this idea was anticipated by vacuolation experiments on aging *Neurospora* mycelium (23, 24), contemporaneously with the early studies of autophagy in yeast and mammalian cells (5, 6).

## RESULTS

### Starvation activates at least two separate but convergent recycling pathways

#### I. Formation of vacuoles (vacuolation)

**Fig 1:**
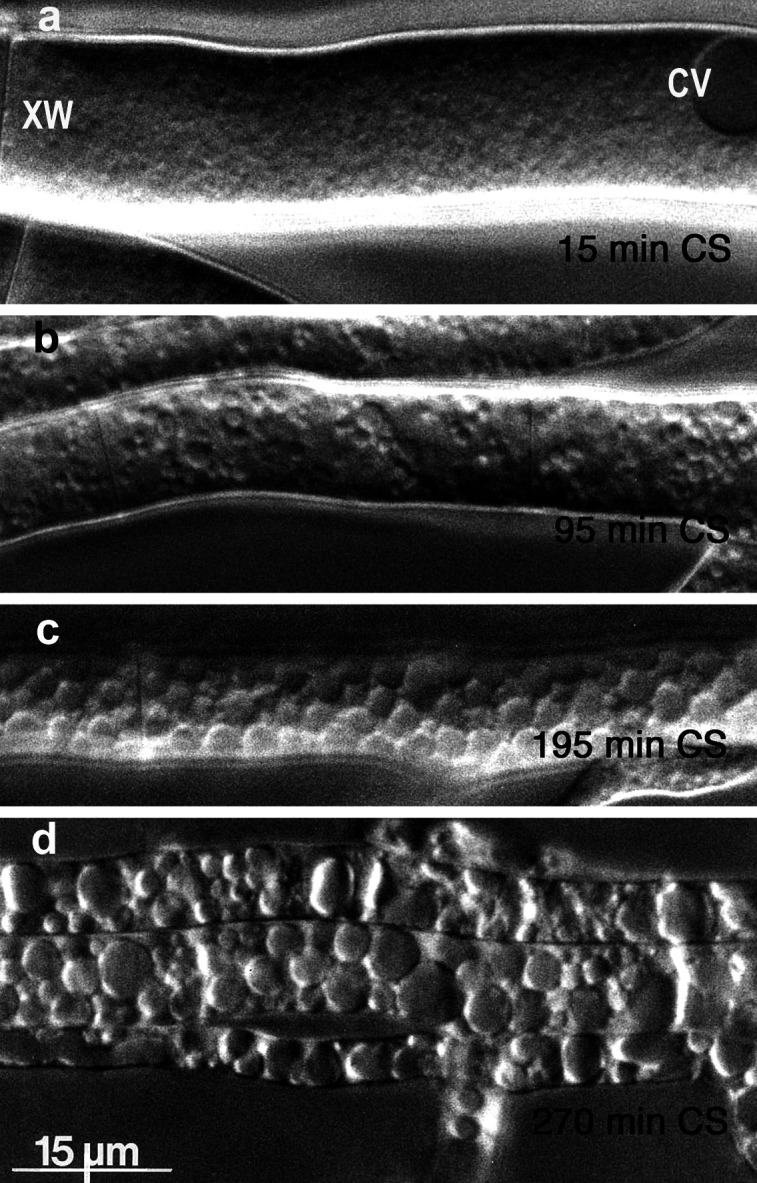
Profuse production and growth of vacuoles within *Neurospora* hyphae, during carbon starvation*.* Panel a: Cytoplasm perceptibly granulated (630x), representing the onset of vesicle enlargement, after 15 min of carbon starvation (CS). Panels b–d: Lengthening starvation times, with visualization by the differential interference contrast (DIC) microscopy. Prior to carbon starvation, *Neurospora* stem hyphae typically possess a single spherical constitutive vacuole (CV) in every second cell. Gossamer mycelium was grown and prepared as described in Methods. In separate experiments, control cells in carbon-replete conditions (1% glucose) did not vacuolate up to 6 h incubation if the gossamer was handled gently. (XW) cross wall. (*Neurospora*’s crosswalls are perforated, allowing continuous cytoplasmic flow for several centimeters in a mature colony. The flow here was rightward.) Strain: RL21a, Rockefeller-Tatum wild type.

**Fig 2:**
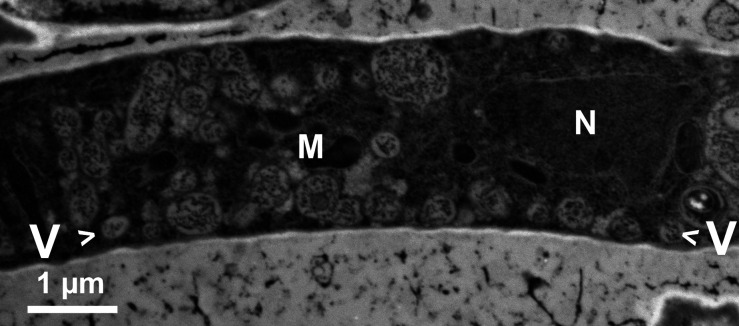
Transmission electron microscopy (TEM) display of small vacuoles, arrayed along the PM of a typical seeker hypha, after 3 h carbon starvation*.* Vacuoles filled with textured material following HPF, freeze-substitution, and staining with Osmium tetroxide (1%) +uranyl acetate (0.1%). Gossamer mycelium was similar to Fig. 1c. Strain eGFP-*ATG8*. (**M**) Mitochondrion, (**N**) Nucleus, (**V**) Vacuole (viewed along the plasma membrane).

Within minutes of the onset of carbon starvation, the observed texture of *Neurospora* cytoplasm turns “sandy,” as viewed by ordinary bright-field microscopy. The progressive change is most obvious in “stem” hyphae (those ~10 µm diameter or larger) but can also be seen as serial granularity in the abundant small “seeker” hyphae. The sandy or grainy appearance is accentuated by differential interference contrast microscopy (DIC), as shown in Fig. 1, panel a. By 20–30 min of carbon starvation, small but frank “*de novo*” vacuoles blossom seemingly everywhere, an appearance which is sensitive to the focal plane, thus, suggesting preferential formation along the cell wall. Indeed, with longer starvations, *de novo* vacuoles enlarge and pack against the plasma membrane, as shown via DIC in Fig. 1b (95 min) and 1 c (195 min); via TEM (transmission electron microscopy) in Fig. 2; and via confocal microscopy in Fig. S2.

**Fig 3:**
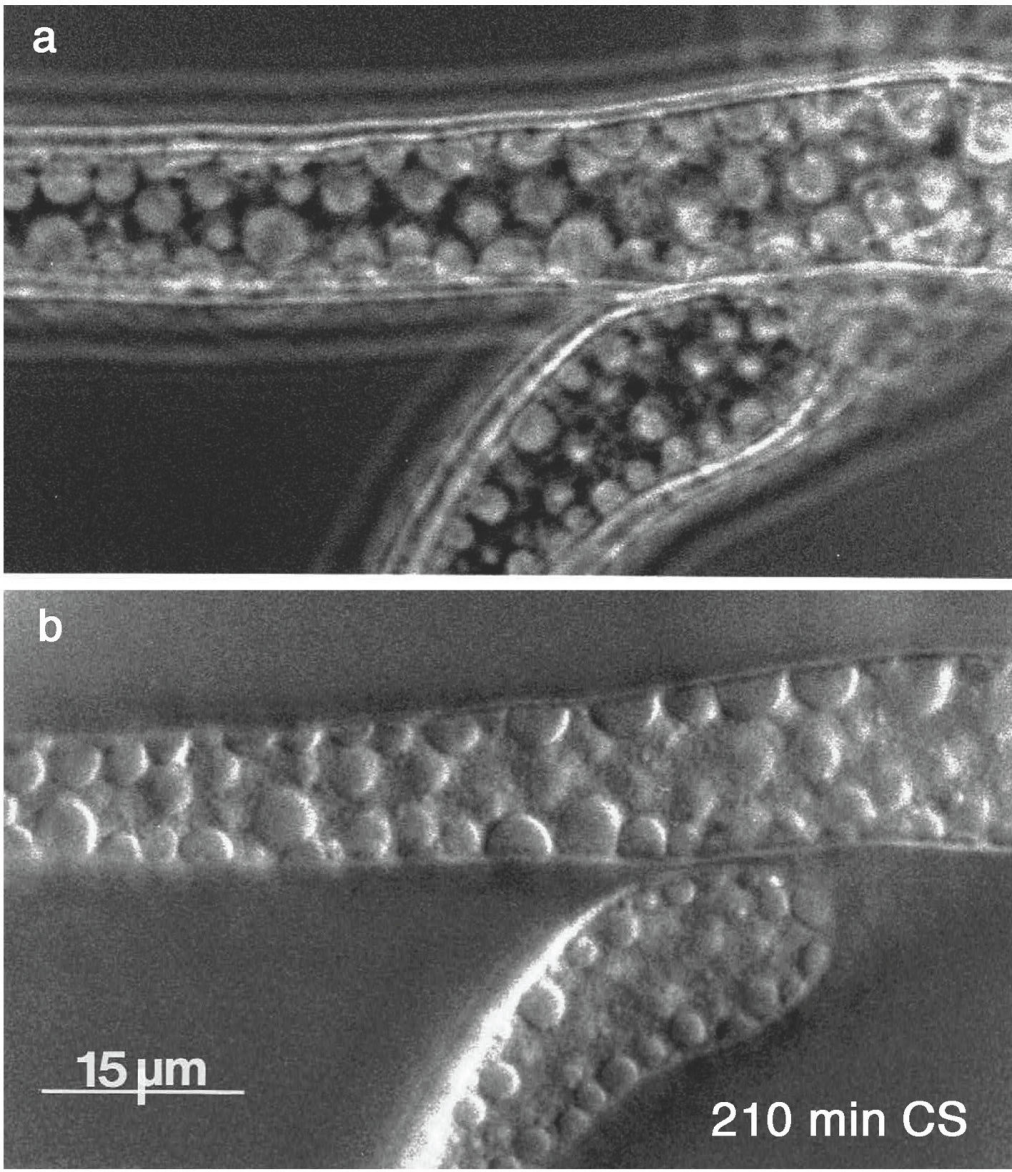
Radial display of vacuoles elicited by C-starvation, with phase-contrast image to capture the 3-D effect. Panel a: phase-contrast highlights 3D by revealing interspaces that are hidden by fringes in Panel b: the differential interference contrast (DIC) image. Same experiment as Fig. 1.

But because packing constrained at the plasma membrane defines limited (2D) space, continuing proliferation or enlargement of vacuoles must result in the literal stuffing of the cell volume, as demonstrated in Fig. 1d (270 min). The progress of such stuffing is emphasized in Fig. 3, by the pseudo-3D appearance of the phase-contrast image (Fig. 3a).

After long starvations, *de novo* vacuoles can come to occupy up to 70% of cytoplasmic volume (see Fig. 1d), and sharp mechanical agitation can trigger their fusion into giant vacuoles. Because overall cell volumes seem unchanged during carbon starvation, expansion of intravacuolar volume requires equivalent movement of water from the cytosol, which would be an automatic physical occurrence when the vacuoles are proteolytic.

**Fig 4:**
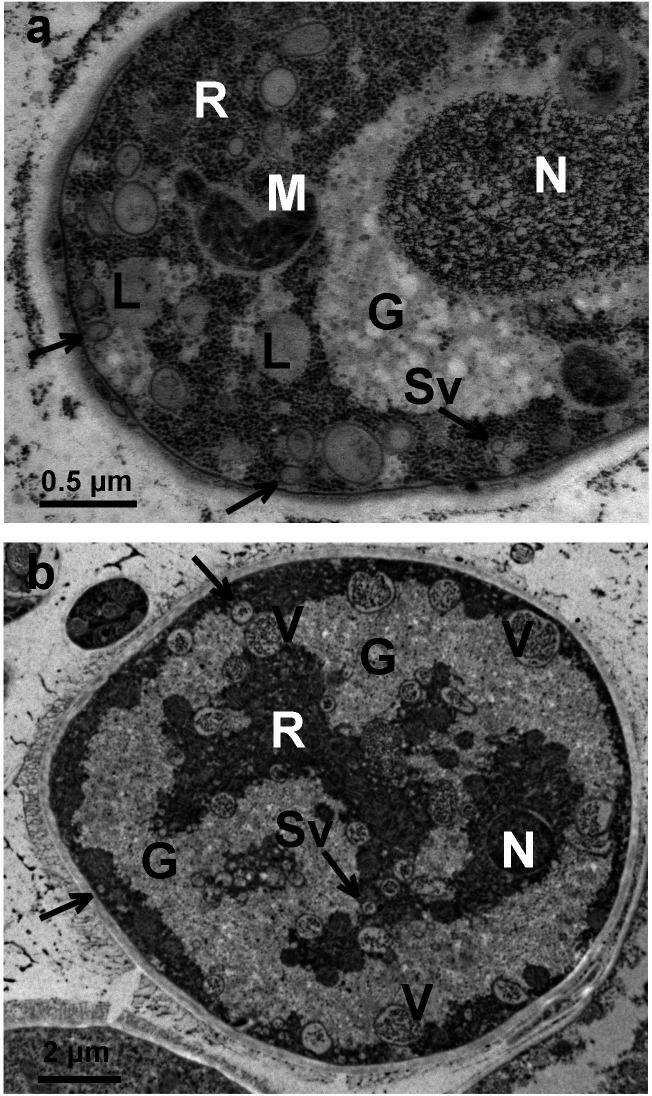
Small vacuoles and vesicles distributed near the PM of carbon replete *Neurospora*. Panel a: Transmission electron microscopy (TEM) image: crown section of a small (seeker) hypha, showing membrane-adjacent vesicles down to 70 nm diameter (Sv, arrows). Ribosomes (**R**) form a punctate background; gray and whitish areas (**G**) are glycogen storage droplets; unbounded gray objects (**L**) are presumed lipid droplets. Panel b: TEM cross-section of a large (stem) hypha, showing bona fide vacuoles (**V**) with textured material contents, plus nearly empty vesicles down to 200 nm diameter. Gossamer mycelium was grown and handled as in Fig. 1 but maintained in 0.5% glucose (28 mM), sufficient to suppress vacuolation. For EM, gossamer was plucked from the watch crystal with jewelers’ forceps, processed through high-pressure freezing (HPF) as described in Methods, then stained, embedded, and sectioned. Strain: eGFP-*ATG8*.

**Fig 5:**
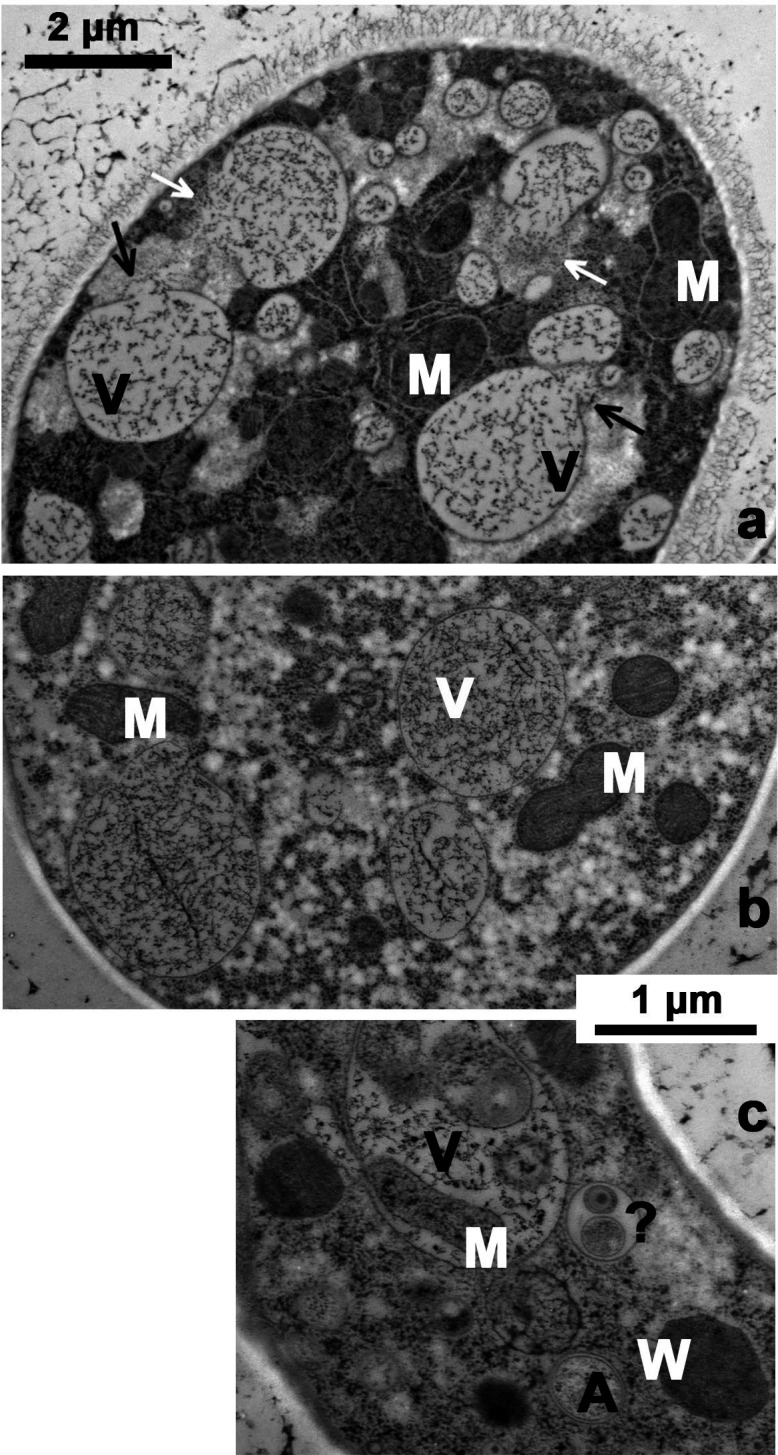
Varied appearance of vacuoles produced during carbon starvation*.* Panel a: Stem hypha displaying vacuoles of 0.2 to ~3.0 µm diameter, after 25 min carbon starvation. Arrows point to vacuolar “tongues” extending into the surrounding cytoplasm, with very indistinct boundary surfaces. This could represent a form of *micro*autophagy, or a mechanism for releasing proteolytic fragments into the tip-ward hyphal stream. Panels b, c: Seeker hyphae having vacuoles of varied content and density, after longer starvation: 75 min and 200 min, respectively. Labels (**M and V**) as in Fig. 4. (**a**) phagophore; (**W**) Woronin body/peroxisome; (?) ill-defined phagocytic body. Vacuole in Panel c contains a mitochondrion and several partially digested organelles. The intravacuolar presence of organelles probably represents *micro*autophagy (25). Gossamer mycelium was grown and handled as in Fig. 4. Strains: wild-type strain OR-74A in panel (a); eGFP-*ATG8* in panels **b**, **c**.

Two possible primordia for the starvation-driven vacuolar profusion in Fig. 1c and d are the pre-existing small vacuoles, plus a native population of still smaller vesicles lying very near the plasma membrane of carbon-replete hyphae. See Fig. 4 panels a, b. The cytological appearance of these vesicles/endosomes seems to depend upon their size, somewhat on the duration of carbon starvation, and—of course—on the methods of fixation and staining applied after trapping by HPF (see Methods). After staining by osmium tetroxide, uranyl acetate and lead citrate, the smallest vesicles in carbon-replete cells (<150 nm diameter) appear empty, whereas larger ones (~300 nm and greater) are filled with lightly textured material, probably representing partially digested protein which has been fixed and stained post HPF. A similar appearance is seen in *de novo* vacuoles of carbon-starved cells (Fig. 5, panels a, c), but a survey of starved cells has revealed widely varied vacuolar contents: scrambled textured material and purloined organelles (Fig. 5c) (25).

**Fig 6:**
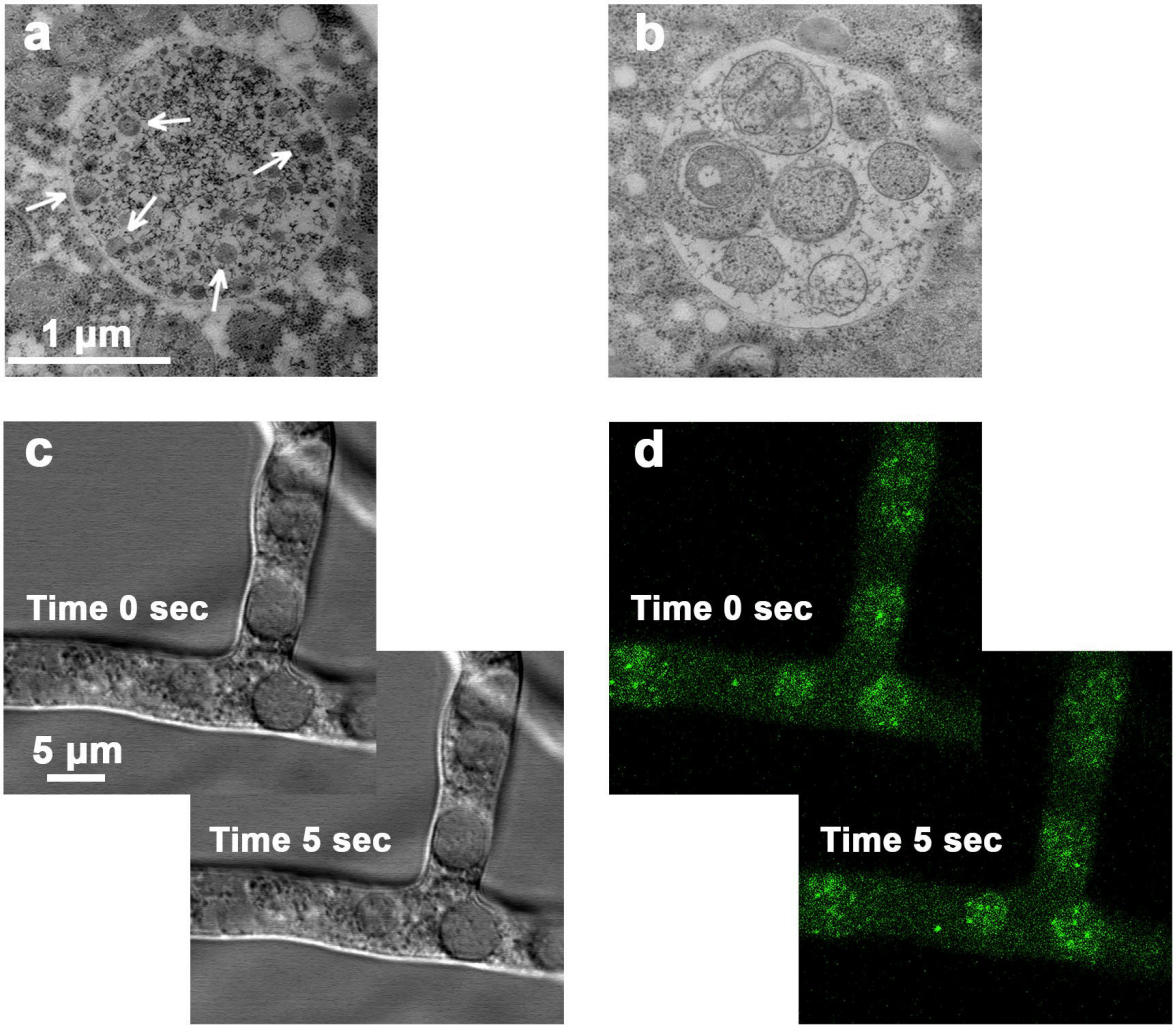
Stabilization of vacuole-trapped proteins and organelles by deletion of ATG15***.*** Panels a, b: TEM of single vacuoles in two different hyphae, showing residual particles of very different size, which implies variable lytic (digestive) action. Arrows in Panel (a) point to some small vesicles. Panels (c) & (d): *Paired images chosen 5 s. apart* to demonstrate cytoplasmic motion by repositioning of individual intra-vacuole particles within that sampling interval. DIC and fluorescent particles mostly colocalize. Note very different scales in panels (a and b) compared with (c and d). A detailed view of the motion is shown in Supplemental Movie 1. Untagged strain showed negligible intrinsic autofluorescence. Mycelia were carbon-starved for 2 h as described in Methods.

In *Saccharomyces*, but not in *Neurospora*, blockade of vacuolar proteases stabilizes the microscopic picture of partially digested vacuolar contents (7). Here, we have focused instead upon *ATG15* (NCU06436), whose yeast homologue is a vacuolar-resident lipase facilitating the breakdown of autophagic bodies, lipids, and organelles (26, 27). After deletion of *ATG15*, *Neurospora* hyphae load up with intravacuolar (IV) fragments, as demonstrated via TEM in Fig. 6a and b. In some cases, the IV fragments are compacted into ~100 nm "dots" amid the textured material protein (panel a). But in other cases, the IV fragments are swollen, like simple autophagic bodies in yeast (panel b). When visualized via light microscopy: DIC or fluorescence (Fig. 6c or d), these IV fragments are seen in Brownian motion, sometimes tethered as if on membrane strings. Detailed visual comparison of the quasi-duplicate images in Fig. 6c or d shows the motion; and cross-image viewing paints fluorescence onto the corresponding DIC objects.

**Fig 7:**
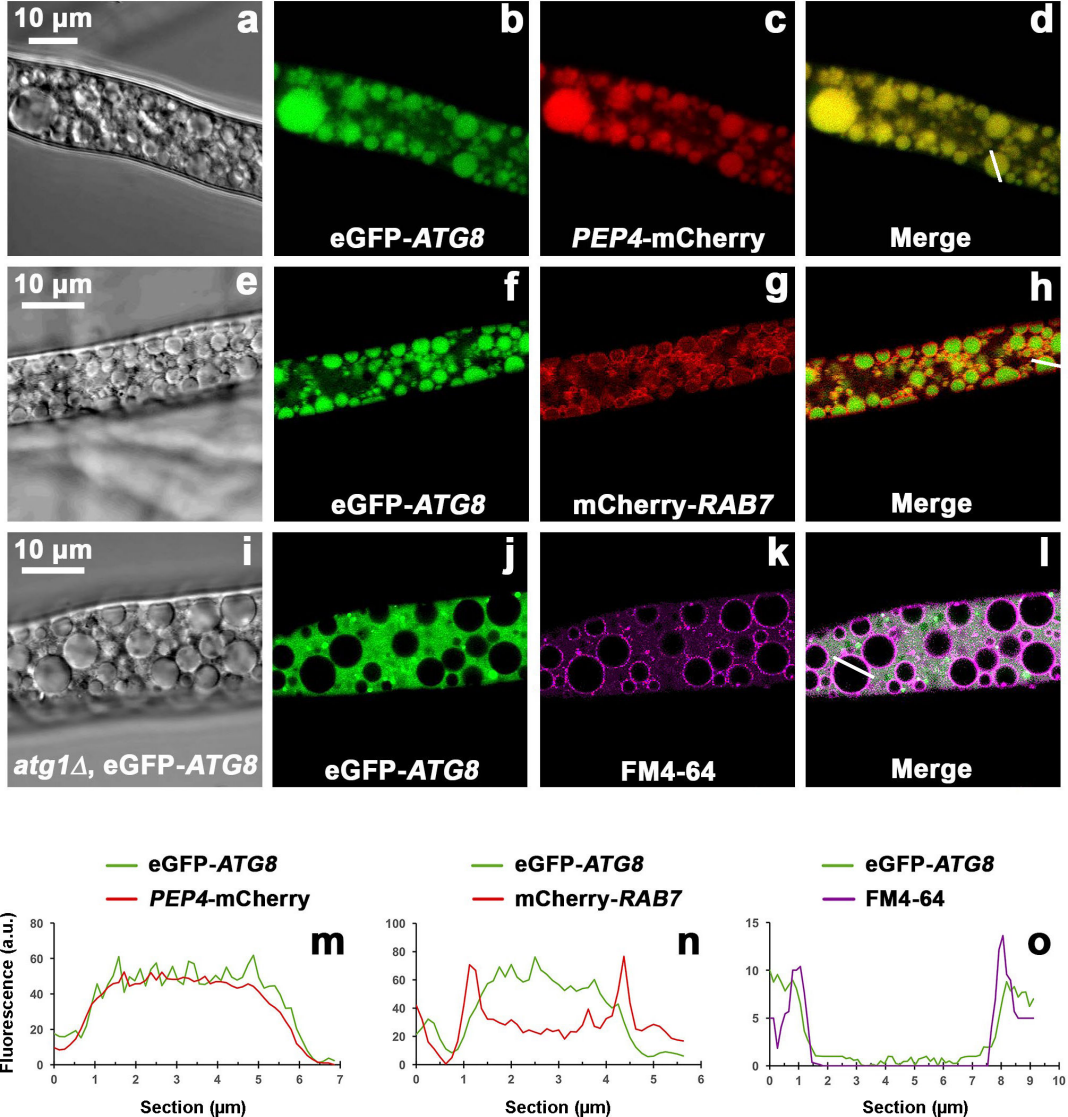
Localization of a few "key" enzymes in *Neurospora*’s pathway for autolysis. Panels a-d: strain expressing *ATG8* tagged with eGFP (N-terminus) and *PEP4* (vacuolar peptidase; mCherry tag to C-terminus). *ATG8* & *PEP4* both enter the whole vacuolar space. Panels e-h: strain expressing e-GFP-*ATG8* and *RAB7* (small GTPase, late endosome marker; mCherry tag to N-terminus). *ATG8* fills the vacuoles while *RAB7* distributes to vacuolar membrane. Panels i-l: strain knocked out for *ATG1* gene (*atg1*Δ), expressing eGFP-*ATG8*, and stained with FM4-64 (a dye for vacuolar membrane). *ATG8* is blocked out of the vacuoles by deletion of *ATG1*. Panels m, n, o emphasize fluorescence intensity distribution along digital sections drawn across a single vacuole (see white lines in merged images). Mycelia of all strains were carbon-starved for 2 h. Scale bars in DIC images (10 µm) apply to the corresponding horizontal image set. Negative controls (non-tagged strains) show negligible autofluorescence.

The major materials stored in *Neurospora* vacuoles are amines, basic amino acids, polyphosphates, and calcium (28). The list goes on, but of prime interest here are several lysosomal proteins displayed in Fig. 7 by means of fluorescent tags: (i) the *presumed* autophagic marker Atg8, filling the interiors of both *de novo* and constitutive vacuoles (panels b & f); and similarly, (ii) the aspartyl protease Pep4 (panel c). In contrast, (iii) the small GTPase Rab7—a classic late endosome-lysosome marker—appears concentrated in tonoplasts/vacuolar *membranes* (panel g), but clearly is *not* in the vacuolar bulk. Coordination of these proteins is discussed below, but two surprises arise: first, constitutive vacuoles and *de novo* vacuoles look the same; and second, Atg8 (Gfp-tagged) appears throughout cytoplasm and in vacuolar bulk, but preferentially *not* in tonoplasts. However, removal of the *Neurospora* Atg1 protein (deletion of gene *ATG1*, NCU00188) excludes Atg8 from the vacuoles (panel j), as had been expected from the known roles of the yeast homolog in recruitment to autophagosomes (7, 9).

#### II. Formation of phagophores (Phagophorulation)

##### Transmission electron microscopy

Autophagy is well studied in yeast (9) and the process is thought to be in large part conserved in other eukaryotic organisms (29). This assumption and massive data collected from yeast have framed yeast autophagy as “canonical,” both morphologically and biochemically (30).

**Fig 8:**
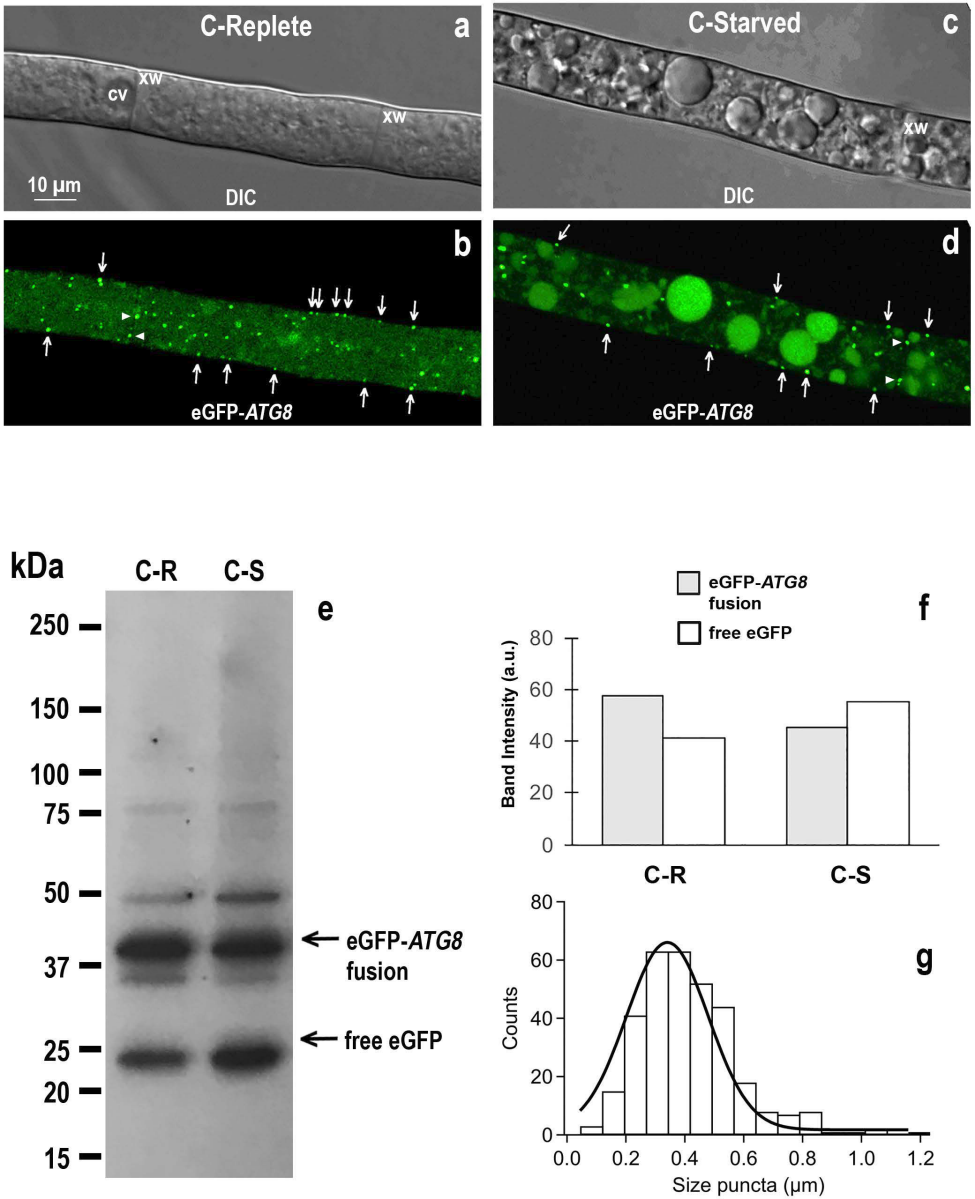
Autophagy indicators in *Neurospora* under carbon-replete and carbon-starved conditions. Mycelial gossamers were incubated for 2 h in the presence of 1% glucose (C-Replete, panels a, b) or zero glucose (C-Starved, panels c, d), mounted on a slide, and immediately imaged using confocal microscopy. Homokaryotic strain expressing *ATG8* tagged with eGFP at the N-terminus. Fluorescent puncta are interpreted as autophagosomes/phagophores carrying lipidated eGFP-*ATG8*. These puncta are present in carbon-replete as well as in carbon-starved *Neurospora* cells. Arrows indicate fluorescent puncta close to the plasma membrane. Arrowheads indicate puncta close to the cross walls (xw). Constitutive vacuole (cv). Scale bar in panel a applies to all four images. Panel e: autophagy flux measured as degradation of eGFP-*ATG8* to free eGFP by vacuolar proteases of replete vs. starved mycelial gossamers; Western Blot of crude total protein extracts (2.5 µg protein/lane) probed with a GFP antibody. Minor crossreacting bands most likely represent intermediate degradation or aggregation products. Western blot is representative of four independent experiments that gave similar results. Band intensities from Western blot in panel e were quantified in panel f using ImageJ software. Panel g: size distribution of eGFP-*ATG8* fluorescent puncta (Gaussian fit). Diameters of quasi-circular puncta were measured using ImageJ and raw data binned (326 total measurements from 10 cells, 16 bins set at 0.074 µm width, average ~0.34 µm).

Although *Neurospora*’s genome contains many atg gene homologs, the real function of *Neurospora*’s actual autophagic organelles (phagophores and autophagosomes) is unknown. To approach this problem, we used a strain expressing eGFP-*ATG8* at the native locus, and inspected mature hyphae for eGFP fluorescence ±glucose (Fig. 8, Panels a,b and c,d, respectively). Both conditions yielded intense fluorescent pinpoints throughout the cytoplasm, but especially at the cell edge. The size distribution of bright fluorescent dots spans from 0.05 μm to 1.2 µm, averaging 0.34 µm (Fig. 8g).

In yeast expressing eGFP-*ATG8*, bright puncta have been interpreted to reflect enhanced fluorescence intensity from lipidated eGFP-*ATG8* (eGFP-*ATG8*-PE), decorating both membranes in nascent and mature autophagosomes (31). We have no reason to reject this view in *Neurospora*, but because the corresponding objects are elusive in TEM images, we do not know for certain whether *bona fide* autophagosomes or intermediate structures glow as the fluorescent pinpoints. Immunogold cytochemistry and correlative light electron microscopy techniques both failed to clearly answer this point, mainly because of background issues. Functionally, however, glowing eGFP-*ATG8* puncta even in carbon-replete conditions implies that *autophagy is a background process* in *Neurospora*. For further testing this idea, we used the eGFP-*ATG8*-expressing strain to measure autophagy flux: how fast autophagosomes are degraded in C-Replete or C-Starved cells. The readout of this assay is the amount of free eGFP (fluorescence) left after attack on eGFP-*ATG8* by vacuolar proteases (32, 33), shown in Western blot analysis in Fig. 8e and f. The band intensity ratio free eGFP/eGFP-*ATG8* fusion is 0.76 in carbon-replete versus 1.26 in starved conditions (Panel f). Thus, *Neurospora* autophagy is abundantly present in carbon-replete cells, but is further induced by carbon starvation.

TEM micrographs clearly demonstrate that *Neurospora* produces a variety of multimembrane autophagy-related structures (phagophores and autophagosomes). These structures arise predominantly from the plasma membrane, rarely—if ever—from the ER. They can be lytic in character, thus clearly differing from the yeast picture.

**Fig 9:**
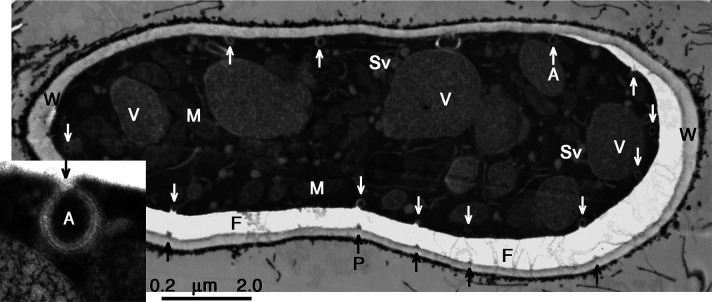
Phagophores proliferated around a single hypha*.* Cytoplasm fortuitously shrunken either during HPF or during fixing & staining. PM thus torn from the cell wall revealed multiple forming phagophores: designated by A & white arrows. A robust plug (or anchor) remained attached to the wall opposite each phagophore (*P* & black arrows), while a corresponding “mouth” gapped the cytoplasm. Slender fibers (**F**) along the gap: part of the severed membrane-to-wall attachments. Labels (V, Sv, & M) as in Fig. 4 and 5. Wild-type strain RL21a. Mycelium carbon starved for 4 h. Inset: Detail of one forming phagophore (**A**). The enlargement of phagophore-to-membrane-wall boundary reveals a faintly bilobed structure in the contact mouth. Scale bar: 2 µm for main, 0.2 µm for inset.

Presumptive simple phagophores, with concentric dual membranes, are shown in Fig. 9, ringing a hypha, attached to the plasma membrane itself (see inset) or having been stripped from an anchor on the adjacent cell wall. In favorable hyphae, phagophores form at intervals of 1.5–2.0 µm along the plasma membrane. That distribution implies 60–70 forming phagophores over the whole cylindrical surface (here, ~15 µm long and 5–6 µm diameter), viz. approximately one for every 5–6 µm^2^ of hyphal surface.

**Fig 10:**
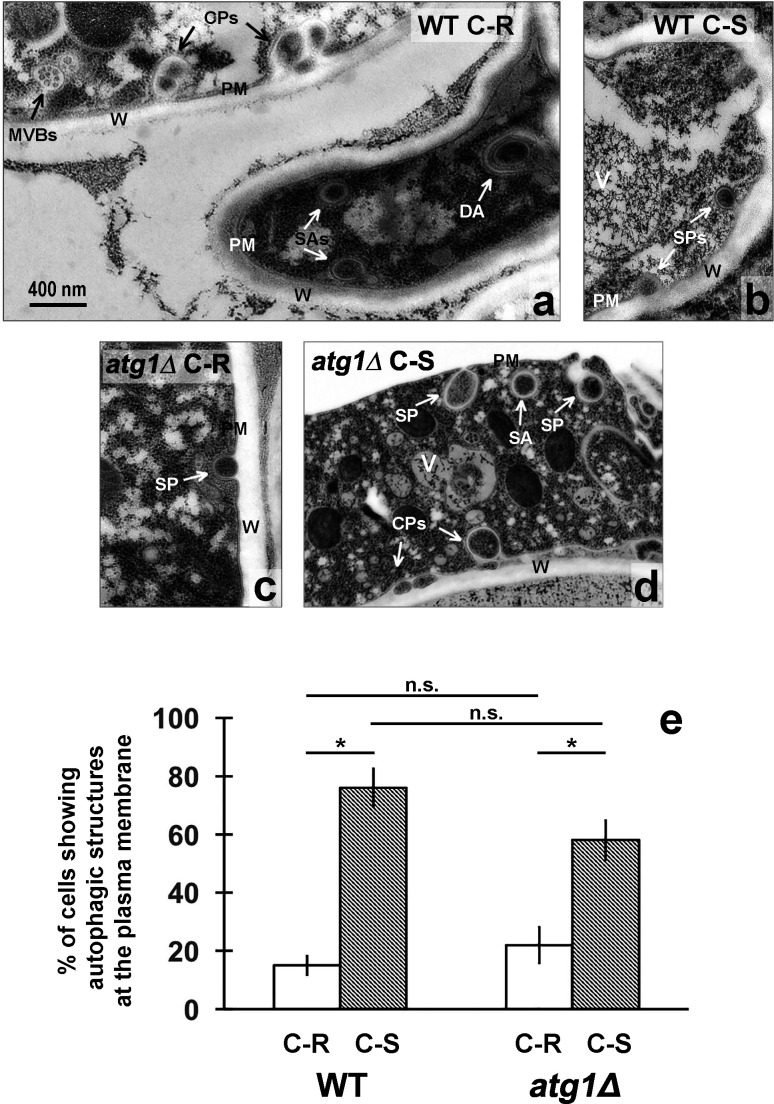
Formation of autophagic structures at the plasma membrane of *Neurospora* increases after carbon starvation and is independent of ATG1*.* Mycelial gossamers of WT and *atg1*Δ strains were incubated for 2 h in the presence of 1% glucose (C-R, Carbon-Replete) or without glucose (C-S Carbon-Starved), harvested, subjected to high-pressure-freezing (HPF) plus freeze-substitution, stained with 2% uranyl acetate +lead citrate and observed using electron microscopy. *ATG1* is a serine/threonine protein kinase which plays a key role during autophagosome biogenesis in yeast, by recruiting the molecular machinery in the phagophore nucleation process. Panels a ,b, c, d: representative TEM images of cells showing autophagic structures still attached to the plasma membrane: (SP) single phagophore; (CP) compound phagophore), or detached from the plasma membrane but close to it: (SA) single autophagosome; (DA) double autophagosome. In panel d, the cell wall detached from the plasma membrane during HPF, leaving the membrane attached to the cytoplasm. (**W**) cell wall, (PM) plasma membrane, (MVBs) multivesicular bodies. Scale bar in panel a (400 nm) applies to all four images. Panel e: quantification of whole data sets represented in panels a-d. Cells were observed by inspecting random fields of view (six per condition) and scoring as positive cells, those showing autophagic structures: at least one phagophore forming at the plasma membrane or at least one autophagosome close to the plasma membrane. Positive cells usually showed two or more autophagic structures. Cells devoid of autophagic structures were scored as negative. Number of cells observed: WT C-R = 101; WT C-S = 41; *atg1*Δ C-R = 41; *atg1*Δ C-S = 48. Error bars indicate ±SE for the indicated proportions. Statistics: pooled z-score tests. (*) Statistically significant at *P* < 0.05; (n.s.) not statistically significant at *P* < 0.05.

More of those autophagic objects are shown in the TEM micrographs of Fig. 10a through d: single phagophores, compound phagophores with multiple double layers of membrane; single autophagosomes, and double autophagosomes. The morphology of these objects can be appreciated by higher magnification (see below) even though full appreciation of 3D structure is limited by the 2D nature of TEM thin sections. Whether the formation of autophagic structures, or their increase by starvation, in *Neurospora,* uses some of the canonical machinery inferred from yeast is unresolved. To investigate this question, carbon-replete and carbon-starved mycelia have been observed via TEM and scored for the *fraction* of positive cells: those displaying phagophores and/or autophagosomes, out of the total cells inspected (Fig. 10a and b representative images; e left, quantification). Although autophagic structures are significantly more abundant in carbon-starved mycelium, blocking canonical autophagy by deleting the *ATG1* gene yielded no useful results (Fig. 10c and d; e right). Clearly, formation of autophagic structures in *Neurospora* does *not* require *ATG1* function, although *Atg1* protein could have a downstream role in delivering autophagic organelles to vacuoles (34, 35).

**Fig 11:**
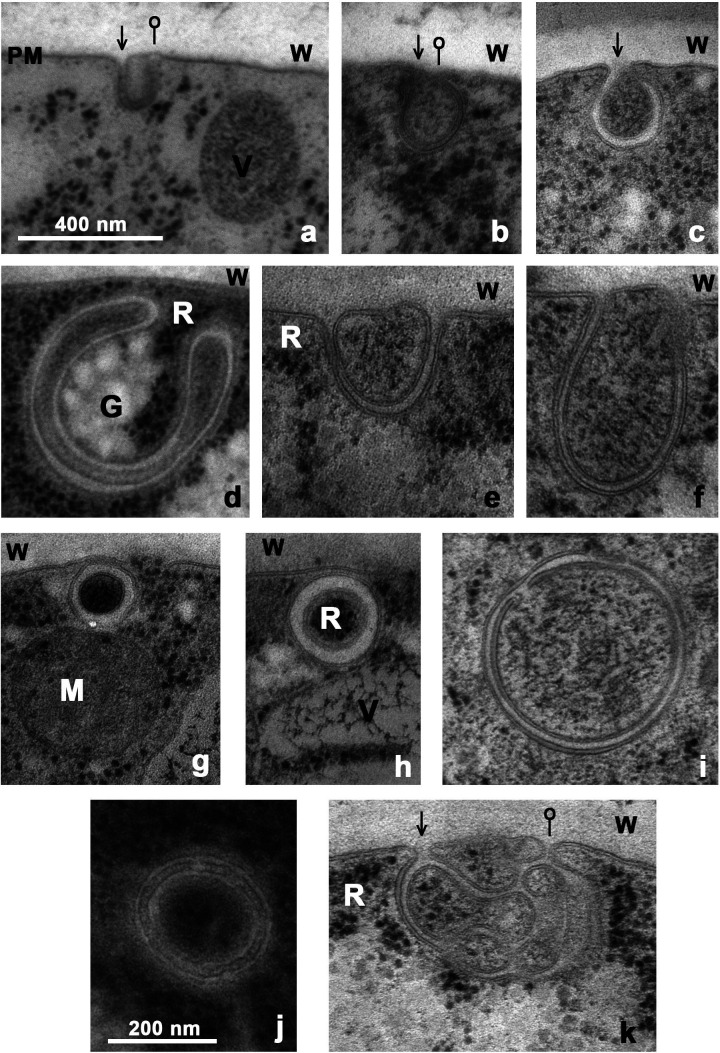
Diverse morphologies of *Neurospora* phagophores*.* Panels a–c: Inward-growing sheets of plasma membrane (arrows), which subsequently curve back toward the PM (pins). Panel d: large intact phagophore with “classical” shape, but with four membranes, detached from PM and completely surrounded by organelles: R (ribosomes) and G (glycogen clumps). Panels e, f: Two phagophores stretching down from PM, apparently filled with ribodigestate. Panel i: similar to e & f, but completely detached from PM. Panels g, h, i & j: similar small phagophores resembling canonical autophagosomes, but h is squeezed between PM & tonoplast, not fused to either; **g** is still fused to PM and pushed against a swollen organelle (likely a mitochondrion); i is already detached from the cell membrane, but shows an open inner membrane; **j** appears free floating, but its two membranes are sewn together by a ladder-like structure. Finally, **k** has been formed by inward growth of at least two visible PM sheets (arrow), then likely reconnection of both (pin) after an internal lytic process, overall resulting in at least five core spaces containing variously degraded ribosomes. Scale bar for panels a-i, k is 400 nm. WT strain carbon-starved for 2 hr. (**w**) cell wall.

High magnification TEM micrographs show how these dynamic organelles evolve starting from the plasma membrane. Phagophores are initiated by inward growth of plasma-membrane (arrows in Fig. 11a through c), which must be sheet-like in three dimensions. The small funnel usually formed at the point of initiation appears filled with cell-wall material (white unstained in Fig. 11a) which could be part of the plug or anchor shown in Fig. 9 and inset. The growing sheets of PM subsequently recurve toward the cell surface, around a gap of tens-to-hundreds of nanometers, before fusing back to the PM (pins in Fig. 11a and b). This action entraps a small volume of cytoplasm which usually contains ribosomes, clumps of carbohydrate, and digested fragments of both. Occasionally a developing phagophore loads up, then detaches from the PM but fails to close, so resembling a canonical phagophore, but with four membranes (Fig. 11d). The obvious dissolution of ribosomes or carbohydrate clumps (Fig. 11g, j and i) seems to report the activity of lysosomal enzymes in the central cavities of the phagophores. Thus, in *Neurospora*, and probably in other mycelial fungi as well, autophagic recycling, downstream trafficking, and tip growth, may be able to proceed without explicit involvement of the actual defined vacuoles.

Compared with the highly detailed but rather rigid picture of autophagosome structure and function in *Saccharomyces*, the morphology of phagophores in *Neurospora* can diverge widely, and is susceptible to some humorous description: “phagosacks” resembling a satchel, ~300 nm deep hung off the PM (Fig. 11e); “phagobags” ~600 nm deep, looking very floppy but pinched to the PM (Fig. 11f); “phagoballs” ~300 nm diameter, packed like a golf ball with ribosomes and either rolled snugly between the PM and an adjacent tonoplast (Fig. 11h), or welded to the PM as well as to a swollen mitochondrion (Fig. 11g). Finally, there are fully “free-floating” phagophores, resembling complete canonical autophagosomes, large (560 nm diam., Fig. 11i) or more compact (310 nm diam., Fig. 11j), and having a ladder-like structure sewn between the two membranes.

*Neurospora* also lays down more complicated geometries for these dual membrane phagophores, as shown in Fig. 11k, where five or more separate cytoplasmic domains are defined by the inward growth and refusion of several sheets of plasma membrane. Clusters of ribosomes are present in some of these domains, but not in others, perhaps due to a collaborating distribution of lytic enzymes.

**Fig 12:**
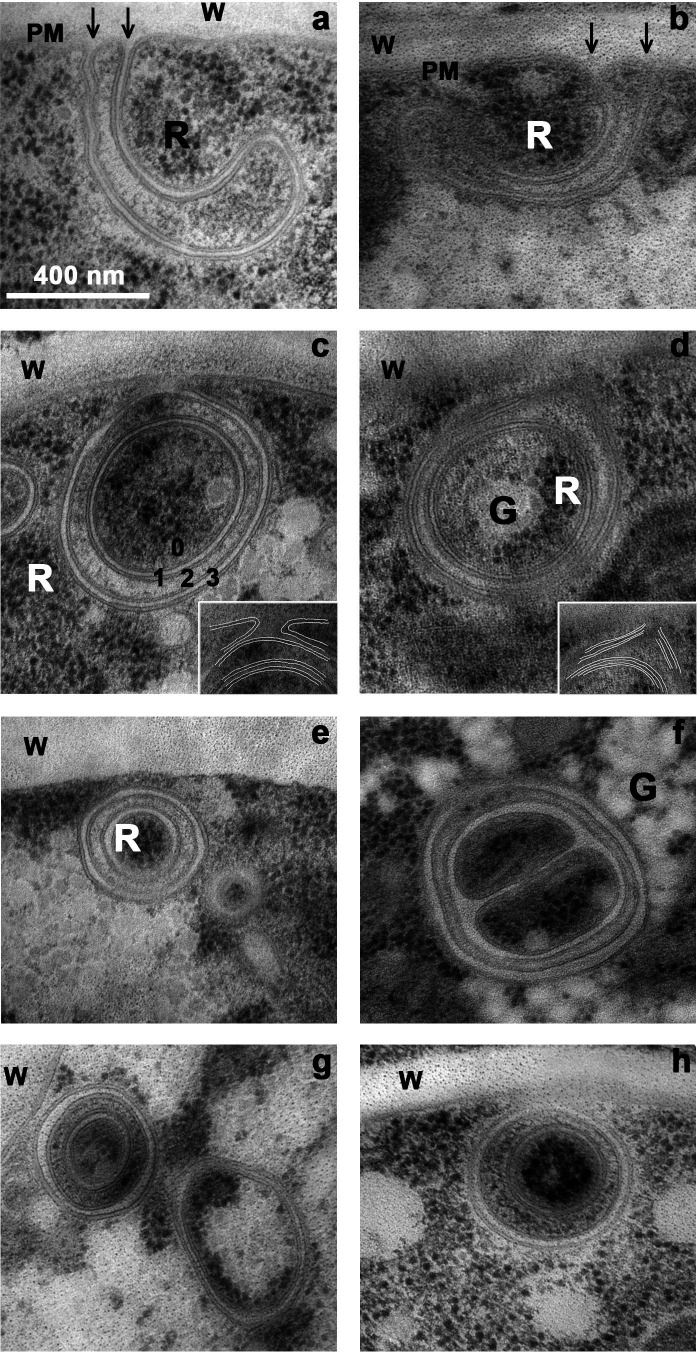
Formation and character of non-canonical double phagophores*.* Panels a, b: Simultaneous in-growth of four sheets of plasma membrane at each panel, fused in pairs before re-connecting to the plasma membrane (PM). (**w**) Cell wall. Panels c, d: double phagophores detaching from the plasma membrane, but with the outer membrane still attached to PM, as shown by both insets. Panel c: Numbered spaces: 0 = main core, 1 = first wrap, 2 = 2 nd core, 3 = 2 nd wrap. Panels e, h, two mature double autophagosomes, cores still loaded with ribosomes, presumably because lysosomal enzymes are missing or inactive. Panel f: Similar to panel c, except that the core space is branched. Panel g: An anemic-looking double autophagosome is paired with a robust single phagophore, the core of which is filled with ribosomes and glycogen clumps. WT strain carbon-starved for 2 h. The scale bar for all panels is 400 nm.

But single phagophores and single autophagosomes (double-membrane structures) are less surprising than double phagophores and double autophagosomes (quadruple-membrane structures). Those are certainly non-canonical since they are rarely observed elsewhere. Double phagophores arise from nearly simultaneous inward growth of two adjacent patches of plasma membranes, shown in Fig. 12a and b, which then curve back to the PM forming *quadruple*-membrane spherical bodies (Fig. 12c through j). The usual clusters of ribosomes and clumps of carbohydrates fill the *central core* of such structures as the inward-growing membranes corral underlying cytoplasm.

To simplify the description of the layered structures, we call the core C0, and count the enclosing shells outward as S1, S2, & S3, labeled as on Fig. 12c. S1 and S3 resemble the outer layer for most single phagophores (Fig. 11f and j) in being generally clear. S2 appears puffy or gritty but is devoid of ribosomes or obvious carbohydrate clumps.

Independent maturation of each phagophore, whether single or doubled, requires several steps away from the PM: (i) actual detachment from the membrane (and anchor); (ii) submersion into the bulk cytoplasm; and (iii) closure of the outer membrane sheath (Fig. 12c-inset, 12d-inset). Phagophores thus separated have been seen squeezed against vacuoles, but we have not caught any actually fusing to vacuolar membranes in *Neurospora*. Although multilayer structures of this sort have a morphological resemblance to the transient so-called “cradles” which support autophagosome formation in cultured mammalian cells (36), single or double non-canonical phagophores usually appear as completely closed structures by 2D imaging, having two, three, or four internal spaces. The inner-most of these *can* contain intact fragments of cytoplasm, including ribosomal clusters and carbohydrate clumps.

**Fig 13:**
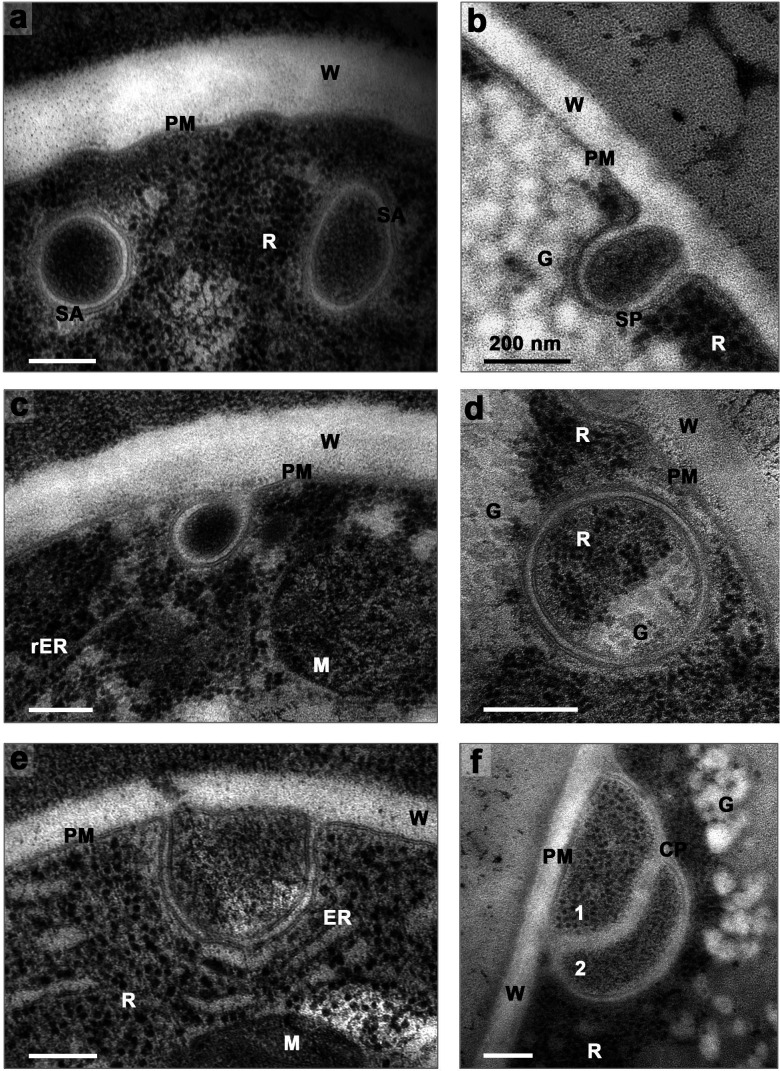
Examples of phagophores and autophagosomes which appear to be lytic*.* The hallmark of these digestive organelles is the absence of defined cytoplasmic components, e.g., ribosomes (**R**) and glycogen clumps (**G**), in their lumen which is instead filled with lysed or partially lysed material. Note the difference between organelle content and surrounding cytoplasm, except in panel d showing a control (non-lytic) single autophagosome containing ribosomes and glycogen clumps which appear identical to those in the adjacent cytoplasm. Panel a: two single autophagosomes (SA) close to the plasma membrane (PM). Panel b: single phagophore (SP) still attached to the PM and devoid of ribosomes and glycogen clumps that are abundantly present in the cytoplasm. Panel c: single phagophore “hook” attached to the PM with partially digested ribosomes. Panel e: single phagophore showing well-digested ribosomes in the lumen. Contact of the phagophore outer membrane with the endoplasmic reticulum (ER), suggests a role of *Neurospora* ER as a source of phospholipids for phagophore expansion (14). Panel f: compound phagophore (CP) outlining two compartments packed together. The compartment close to the plasma membrane (#1) contains ribosomes still intact, while the one further from the PM (#2) contains only digested material. This clear difference between compartments of the same organelle excludes staining artifacts. All images are from WT cells carbon starved as in Fig. 10. The scale bars in every panel represent 200 nm. WT strain carbon-starved for 2 hr. (**M**) mitochondrion, (**W**) cell wall, (rER) rough endoplasmic reticulum.

Notably, the inner cavities of phagophores and autophagosomes often appear devoid of well-defined cytoplasmic components, such as ribosomes and glycogen clumps. This is true in about 40% of the observed autophagic structures in *Neurospora* and strongly suggests a self-sufficient lytic function of these organelles. Fig. 13 shows a few examples of lytic phagophores/autophagosomes (panels a, b, c, e, f), and a non-lytic autophagosome as a control (panel d). Although PEP4-mCherry and eGFP-ATG8 did not clearly colocalize (data not shown), other hydrolytic enzymes could work within these organelles.

### Electron tomography

**Fig 14:**
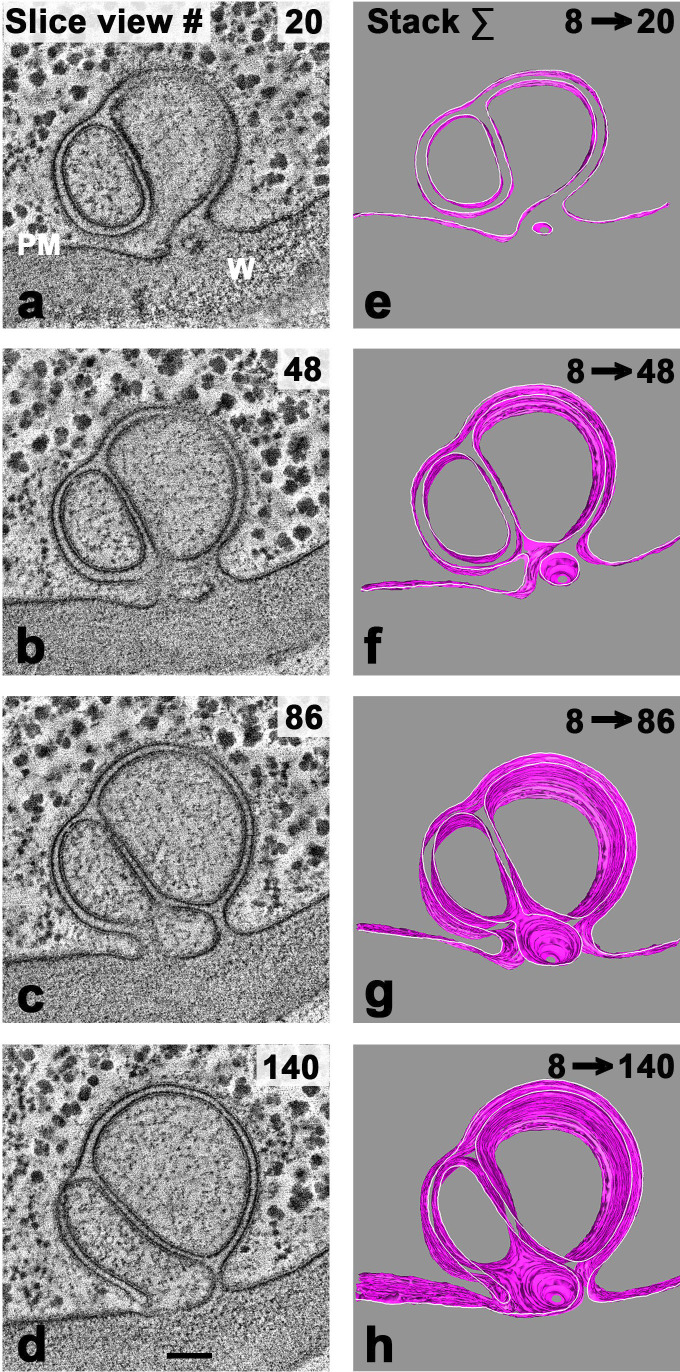
3-D tomographic reconstruction of a branched and twinned single phagophore, growing from the plasma membrane. Left-hand column: Computer-generated plane sections: **a** = “near bottom” to **d** = “top.” Thickness of section images, 1.29 nm. Right-hand column, e to h: gradual construction of 170 nm tall cylindrical structure by stacking of membranes in the top 132 sections, represented by the four calculated sections in panels **a** to **d** (left-hand column). Data obtained by multiply tilted imaging of a thick block, via the same software as in Fig. 15. Stacks 86 to 140 show a clear vertical-space extension between the two membranes surrounding the left-hand cavity. See Fig. S3 for fixed 3D prospectives of these data, for a montage of TEM slice views 8 →140, and ) for full reconstruction of stacks, made through multiple angles. Same gossamer and HPF material as in Fig. 12. Scale bar for all panels is 100 nm. (PM) plasma membrane, (**W**) cell wall.

All of the EM structures shown so far have emerged from TEM thin sections (~60 nm), which reveals the majority phagophores to be circular—or en route to becoming circular—and consisting of two or four separate quasi-concentric membranes. Fig. 11k, however, suggests that *Neurospora*’s phagophoric structures in general may be more complicated than can be revealed by the two-dimensional technique. We therefore moved to tomography (3D) on thick sections (~250 nm), then reconstructed images from tilt series, as detailed in Fig. 14, with a simple two-membrane non-canonical phagophore. Although the structures we could access this way were incomplete, they nevertheless did reveal important complexities.

This phagophore was formed by the inward growth of two parallel PM sheets, similar to those displayed in Fig. 11a through c, e and f. In this case, however, the in-growing sheets did not simply close back to adjacent PM, but branched and partitioned off a second, parallel, internal space before RE-fusing to the PM. (This is a distinction in *space*, not in time.) The data set do not include the top or bottom portions of the whole cylindrical structure, but the midportion can be built up progressively by superposing individual slice views, from 8 to 140, as shown in the right-hand column. Sections 20 and 48 (panels a and b) demonstrate that the large right-hand cavity (still connected to the cytoplasm) is bounded by two layers of plasma membrane. For the smaller left-hand cavity, that fact is not obvious, but the small intrusion at the base (slice views 20 and 48) does become continuous with the left-hand cavity. The two “central” cavities here are nearly devoid of ribosomal clusters from the immediately surrounding cytoplasm, suggesting that lytic enzymes were active throughout formation of this complicated phagophore.

**Fig 15:**
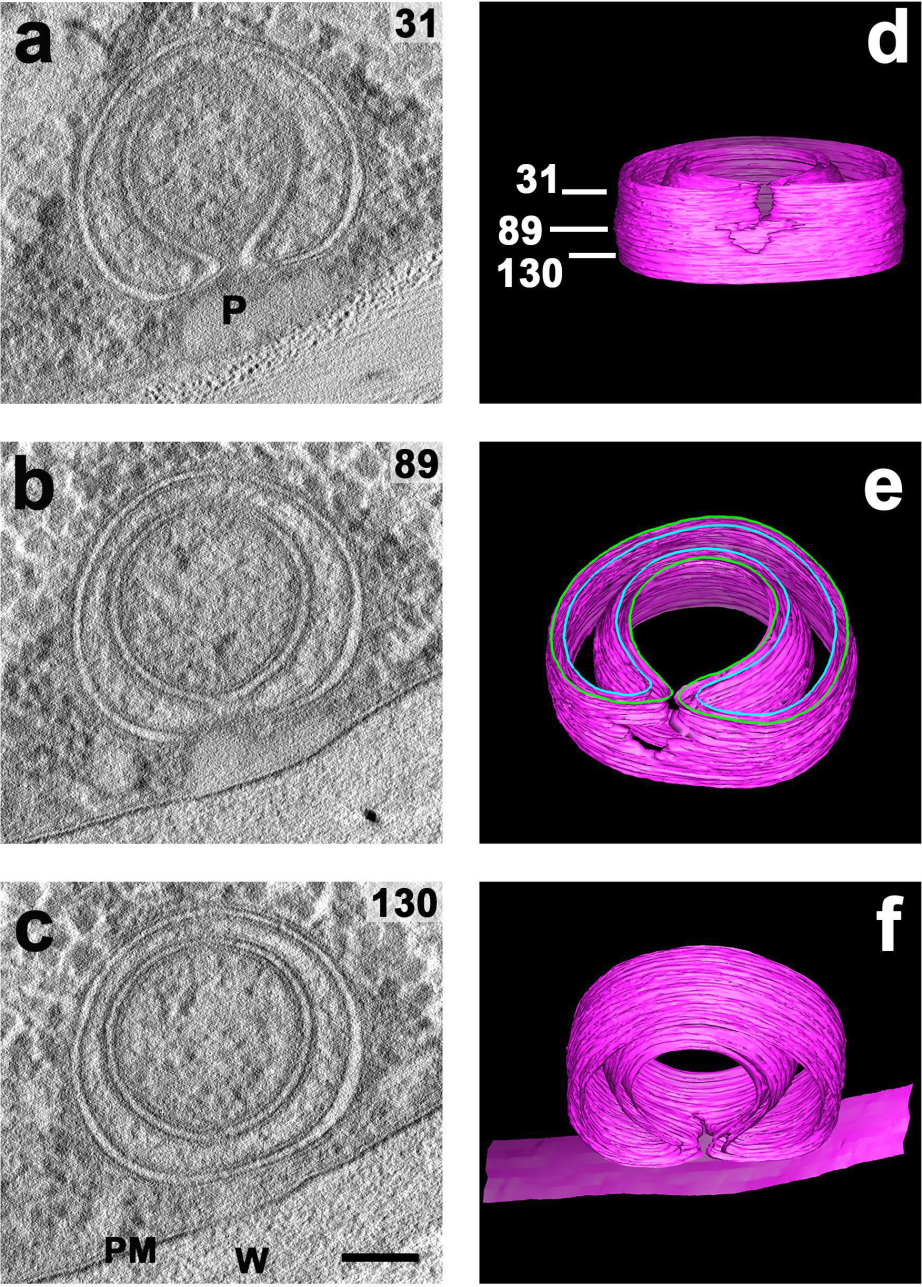
Electron tomographic reconstruction/segmentation of a doubled phagophore in *Neurospora*. Panels a, b, c: Representative slice-views of the tomogram. Panels d, e, f: Segmentation of the same tomogram at different viewing angles (see Methods). Numbers in panel **d** are the distances, in nm, from the top edge of the outer wrap of membranes to the slice-views a, b, & c, respectively. Distance between adjacent slices is 1.108 nm, and the entire tomogram is 153 nm thick (tall). Top contour lines are painted blue/green to emphasize the presence of dual membrane sheets (each contour line represents one membrane bilayer). **P** the non-descript plug (or anchor) against the cell wall, visible in sections a & b, recalls Fig. 9, above. The plasma membrane (PM), which is clear in b & c, has been added to projection **f**. The same gossamer culture and HPF material as in Fig. 12. Horizontal scale bar in all panels is 100 nm. Reconstruction/segmentation carried out via IMOD software (see Methods). (**W**) cell wall.

Tomographic results for a non-canonical doubled phagophore, similar to those depicted in Fig. 12, are displayed in Fig. 15. The resulting structure proved not quasi-spherical, but cylindrical, having two almost complete double-membrane rings around the lower half (near slice-view c), but opening from a discrete focus (plug-like object) against the cell wall in slice-view a. The top of this double cylinder had been truncated by the microtome, but the bottom was left nearly intact, closed (above slice-view c) for the inner cylinder, and imaged (poorly) for the outer cylinder, *as if it lay between* the two membranes. The 3D projection in Fig. 15d displays the whole object almost edge-on, seen outward from the PM. The projection in Fig. 15e shows the object tilted about 60^o^, depicting the upper surfaces of both bottom membrane wraps as background, and the top edges of both membranes highlighted in blue/green. The projection in Fig. 15f shows the inner surface (back wall) of the cavity formed in this double phagophore by the central ring/wrap of dual membranes. It also displays the discontinuities of both double rings pointing toward the actual plasma membrane, as well as the local merger of the two double rings.

## DISCUSSION

The first surprising effect of carbon starvation in *Neurospora* is the enormous vacuolar expansion. That is *not* a conservative process. Filling 70% of a stem hypha with ~3 µm membrane-bounded spheres means manufacturing or collecting about 4- to 5-fold as much membrane as would be present in the starting plasma membrane. Also, if the *de novo* vacuoles in Fig. 1 in fact do arise from the small vesicles shown in Fig. 4, then the needed expansion of surface area can be estimated from the relative mean diameters: less than 300 nm in Fig. 4, and ~3000 nm in Fig. 1d, implying a ~100-fold increase of vesicular surface area. At least two forces would be driving this membrane expansion: osmosis and biochemical regulation. Cytosol and vacuolar fluid must remain in osmotic equilibrium, so cytosolic water would osmose into the vacuoles as organelles and proteins are degraded into small osmotically active molecules, unless and until these small molecules are extruded. For such lysis-induced expansion really to work, the smallest starting vesicles should contain lytic enzymes, meaning that the visible vacuoles grow from pre-existing lysosomes. So, even without stains for cytochemical proof, the smallest vesicles in Fig. 4 should be lysosomes. These might operate autonomously, except for the energy required to build or recruit vacuolar membrane; and for the energy needed to absorb or extrude cytoplasmic components. In fact, small vesicles labeling with vacuolar markers have been widely observed in filamentous fungi and considered to be part of the complicated “vacuolar compartment,” which includes textbook-classic spherical vacuoles as well as interconnected tubular-vesicular structures (37–39). Given the magnitude and speed of vacuolar membrane expansion (Fig. 1), sources for phospholipids feeding sustained tonoplast growth are an interesting and important mystery. Two possibly significant sources are (i) classic vesicular trafficking, which mediates lipid transfer between organelles via vesicle fusion (40) and (ii) bulk lipid transfer at overt contact sites between organelles (14, 41, 42).

The canonical hallmark of autophagy is the formation of so-called autophagosomes, drone-like organelles which engulf cytoplasmic components either randomly (non-selective/bulk autophagy), or specifically via receptor-mediated cargo recognition factors. In yeast and mammalian cells, autophagosome biogenesis involves about 20 autophagy-specific genes/proteins, which make the canonical core ATG machinery (43, 44). This machinery carries out *nucleation*, *expansion,* and *closure* of specialized cup-shaped cisternae called phagophores–a single-membrane element formed by fusion of diverse vesicles (29, 30). The process is assisted by scaffolding proteins, and occurs at the vacuolar membrane in yeast (29), or in mammalian cells, at a discrete region of the ER-termed the omegasome (36).

In *Neurospora*, by contrast, we found that autophagosome biogenesis results in a surprising variety of structures, having shapes far from the conventional idea of quasi-spherical organelles: for example, double phagophores (Fig. 10, 12 and 15), compound phagophores (Fig. 10 and 11), and twinned and branched phagophores (Fig. 14). The origin of these structures is at the plasma membrane, clearly shown by the continuity between the limiting membrane of most phagophores and the plasma membrane (Fig. 11 to 14). Otherwise documented sources include the ER (36, 45), ER-Golgi intermediate compartments (ERGIC) (46), the mitochondrial outer membrane (47), the nuclear membrane (48), and the plasma membrane (49). The nuclear membrane as a source of autophagosomes is particularly relevant because of the morphological similarity between double phagophores/autophagosomes made by macrophage nuclear membranes in response to herpes simplex viral infections (48) and those made by *Neurospora*’s plasma membrane in response to carbon starvation (Fig. 12c, d, e, g and h). Whereas plasma membrane contribution to autophagosome formation is often seen as a mechanism for extra-membrane during autophagic surges (49), *Neurospora*’s plasma membrane builds whole phagophores and autophagosomes *in situ* (Fig. 11 to 15).

The peculiar feature that forming phagophores can have a firm plug on (or anchor to) the cell wall *through* the plasma membrane (Fig. 9 and 15) has not been reported elsewhere. That plug/anchor has an unknown function for single phagophores in *Neurospora*.

In canonical autophagosomes, the space between outer and inner membranes is described as a tight sub-compartment which can be expanded by fusion with lysosomes to allow degradation of inner membrane and resident cargo in a newly formed *autolysosome* (13, 29). In *Neurospora* double and compound phagophores, the picture is more complicated: for example, the intermediate space (#2) between the two dual membranes is devoid of cytoplasmic objects (Fig. 12c through h), implying that *proteases must be activated within* the space captured by advancing pair folds of PM (Fig. 12a and b); also, central cavities of compound phagophores lack cytoplasmic material (e.g., Fig. 13 and 14) strongly suggesting degradative functions.

We do not know whether these organelles carry out their lytic process *instead of* the expected cargo-transport function, or both. However, the conspicuous filling of vacuoles with green fluorescence (Fig. 8d) strongly suggests that cargo-competent phagophores at the plasma membrane must undergo maturation and detachment from the plasma membrane *before* fusing to *de novo* vacuoles during starvation. Thus, robust autophagy flux even in carbon-replete cells (Fig. 8e and f) could be supported by lytic-competent phagophores and autophagosomes, as well as by other components of *Neurospora*’s elaborate vacuolar system (37–39).

The direct involvement of plasma membrane in the manufacture of phagophores and autophagosomes, in addition to the apparent lytic autonomy of those organelles in *Neurospora,* could frame our understanding of how the familiar autophagosomes may have originated and evolved from organisms not yet equipped with a system of endomembranes (50, 51).

## MATERIALS AND METHODS

### Media, strains, genetics, DNA constructs

*Neurospora* strain RL21a, brought from the Tatum collection at Rockefeller University, was maintained in silica-gel cultures at 4°C. Similarly, the Oak Ridge wild-type strains OR-74A and 74-OR8-1a, along with several single-gene KO strains, were obtained from the Fungal Genetics Stock Center (fgsc.net; Department of Plant Pathology, Kansas State University, Manhattan, KS 66506) and stored as silica-gels. All strains could be retrieved from the frozen cultures as needed, and were normally refreshed at yearly intervals, thence maintained on Vogel’s minimal medium (52) +2% sucrose. New constructs usually were started with FGSC strain 9718 a (mus51Δ:: bar^+^), in which *Neurospora’s* robust heterologous recombination is suppressed (53).

Strain eGFP-*ATG8* was constructed by adding a non-dimerizing variant of eGFP) to the N-terminus of *Neurospora’s ATG8* gene (NCU01545). We then constructed four principal strains (Table 1, strains 7–10) all bearing eGFP-*ATG8* as a supposed marker for conventional autophagy. This construct was paired with four other constructs.

**TABLE 1:** Strains used in this study

Strain name	Genotype	Origin
eGFP-*ATG8*-HYG	*eGFP-ATG8-hph*	Transform 9718 a
eGFP-*ATG8*-NAT	*eGFP-ATG8-nat1*	Transform 9718 a
mCherry-*RAB7*	*mCherry-RAB7-hph*	Transform 9718 a
*PEP4*-mCherry	*PEP4-mCherry-hph*	Transform 9718 a
*atg1*Δ	*atg1::hph*	FGSC 17400
*atg15*Δ	*atg15::hph*	Transform 9718 a
eGFP-*ATG8*, mCherry-*RAB7*	*eGFP-ATG8-hph, mCherry-RAB7-hph*	Cross [1] x [3]
eGFP-*ATG8*, *PEP4*-mCherry	*eGFP-ATG8-hph, PEP4-mCherry-hph*	Cross [1] x [4]
eGFP-*ATG8*, *atg1*Δ	*eGFP-ATG8-hph, atg1::hph*	Cross [1] x [5]
eGFP-*ATG8*, *atg15*Δ	*eGFP-ATG8-nat1, atg15::hph*	Transform [2]
9718 a	*mus51::bar*	FGSC
RL21a	*WT*	FGSC 2219
WT74	*WT*	FGSC 2489A

In Strain 7: mCherry-*RAB7*, a late endosome marker; in Strain 8: *PEP4*-mCherry, marking a vacuolar protease; in Strain 9: deletion of *Neurospora's* homologue NCU00188 of yeast ATG1—a kinase involved in early autophagosome biogenesis; in Strain 10: deletion of *Neurospora’s* homologue NCU06436 of yeast ATG15—vacuolar lipase responsible for breakdown of autophagic bodies.

The homokaryotic double-mutants for Strains 7–9 were each obtained by standard crossing of parents bearing the singly tagged genes (Table 1, strains 1–6). However, Strain 10 required more work and was obtained by transformation of an eGFP-*ATG8* strain carrying the nourseothricin selection marker with an *ATG15*-deletion cassette bearing the hygromycin B selection marker.

### Preparations for microscopy

In most experiments, mycelium was grown from an agar-button inoculum on cellophane, underlain by Vogel’s minimal medium plus 2% sucrose and 2% agar. After 18 h at 25°C, a square of cellophane with mycelium attached was cut out and transferred to ~8 mL of Vogel’s medium in a large (6–7”) watch crystal and agitated to float mycelium away from the cellophane. (We call this very thin, free-floating mycelium “gossamer.”) Gossamer mycelium, submerged by repeated droplets of Vogel’s medium, could then be maintained in the growth medium, or transferred (via a coverslip) to glucose-free medium for any appropriate interval.

For light microscopy, the gossamer was picked up on a large coverslip, drained of most fluid, and inverted into a microscope chamber bearing small fluid ports. Thereafter, the chamber was perfused with the appropriate medium and could be viewed by bright-field, phase-contrast, or DIC optics, without staining or fixation, but with continuing solution flow, to prevent anoxia.

For electron microscopy after high-pressure freezing (HPF), the appropriately conditioned gossamer (i.e., carbon-starved or carbon-replete) was plucked “by the tail” with jeweler’s forceps, blotted lightly, then dunked in a drop of hexadecene, removed, blotted lightly again, loaded into a 3 mm brass carrier, and high-pressure frozen using a Leica EM HPM100 at 2100 psi in liquid nitrogen. Frozen samples were freeze-substituted via a Leica Freeze-Substitution unit (AFS2) using the following protocol: 3 h at −95°C in 1% osmium tetroxide/0.1% uranyl acetate in pure acetone; 46 h at −90°C; 20 h at −50°C; then rinsed in acetone over 4 h, to −20°C. Samples were then infiltrated with graded LX112 epoxy resin (Ladd) in acetone over 4 h, to 4°C. After two changes into pure LX112, samples were placed in gelatin capsules and infiltrated under vacuum in pure resin over 2 hr, then hardened overnight in a 60°C oven.

Sections (60 nm thick) were cut using a Leica EM UC7 ultramicrotome, collected onto formvar/carbon coated grids, and stained with 2% uranyl acetate plus lead citrate. Grids were examined on a FEI Tecnai Biotwin TEM at an accelerating voltage of 80 kV. Digital images were recorded with a Morada CCD camera and iTEM (Olympus) imaging software.

For electron tomography, 250 nm sections from the same blocks were cut with the same ultramicrotome, and 15  nm fiducial gold particles were added to the sections before imaging. The dualaxis tilt series was collected via a FEI Tecnai TF20 at 200 kV TEM equipped with a field emission gun. Images were recorded using SerialEM software (UC, Boulder) and an FEI Eagle 4K x 4K CCD camera. Dual-axis tilting angles were from −60° to 60° at 1° increments. Tomogram reconstruction, segmentation, and modeling were performed using IMOD software (54).

Technical note concerning terminology for EM tomography: In Fig. 14 and 15, the TEM-like images (left-hand columns) are computed and represent surfaces only, not objects with depth. They are properly termed “slice views.” However, for the sake of simplicity, we sometimes refer to the slice views displayed as “sections.”

## SUPPLEMENTAL MATERIAL

Fig. S1 msphere.00460-23-s0001.pdf Carbon starvation versus nitrogen starvation.

Fig. S2 msphere.00460-23-s0002.pdf Hyphae ortogonal sections.

Fig. S3 msphere.00460-23-s0003.pdf Phagophore projections.

Movie S1 msphere.00460-23-s0004.avi DIC and eGFP show of *Neurospora* strain *atg15*Δ, eGFP *ATG8* (see Fig. 6).

Movie S2 msphere.00460-23-s0005.avi eGFP show of *Neurospora* strain *atg15*Δ, eGFP *ATG8* (see Fig. 6).

Movie S3 msphere.00460-23-s0006.avi Slice views of tomogram shown in Fig. 14.

Movie S4 msphere.00460-23-s0007.mov Reconstruction of tomogram shown in Fig. 14.

ASM does not own the copyrights to Supplemental Material that may be linked to, or accessed through, an article. The authors have granted ASM a non-exclusive, world-wide license to publish the Supplemental Material files. Please contact the corresponding author directly for reuse.
