# Supplementary material for: Morphodynamics of non-canonical autophagic structures in Neurospora crassa
Source: mSphere. 2023 Oct 17;8(6):e00460-23. doi: 10.1128/msphere.00460-23 (PMC10732065; doi:10.1128/msphere.00460-23)
Supplement: Fig. S1 — Carbon starvation versus nitrogen starvation. [file msphere.00460-23-s0001.pdf]

Supplemental Figure 1

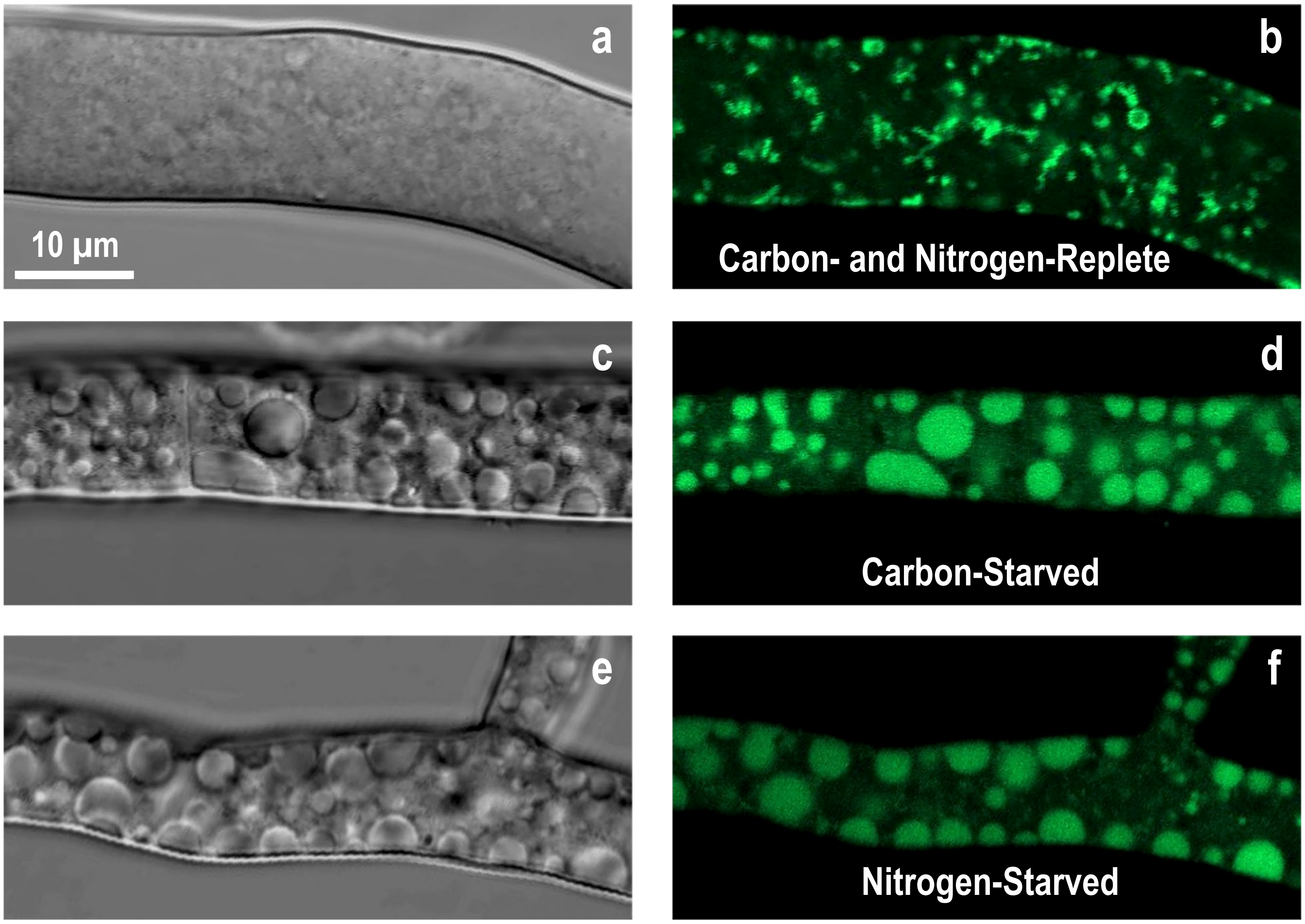

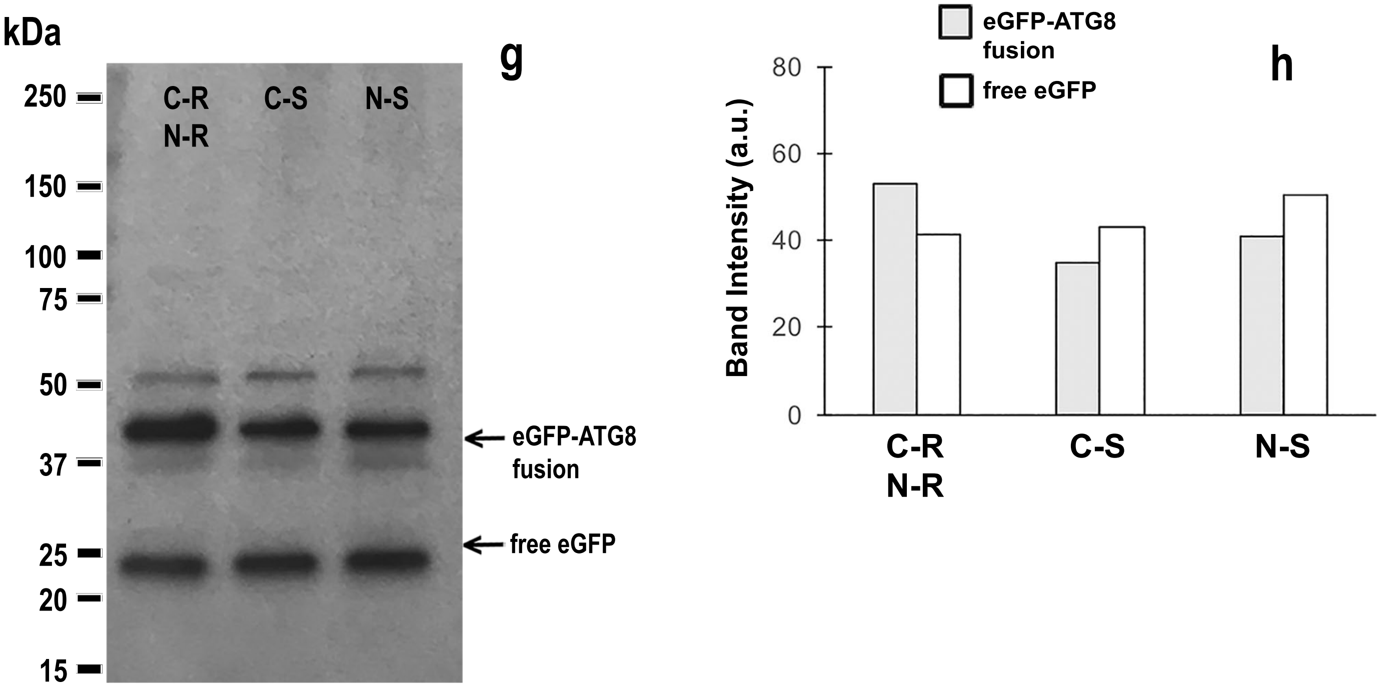

Supplemental Figure 1. ***Carbon-starvation and Nitrogen-starvation induce similar vacuolation and autophagy flux in*** **Neurospora** ***stem hyphae.*** Mycelial gossamers were incubated for 2h at 25C in Vogel's medium (49) + 1% glucose as the sole carbon source (**Panels a,b**: Carbon- and Nitrogen-Replete), or Vogel's medium without glucose (**Panels c,d**: Carbon-Starved), or Vogel's medium without ammonium and nitrate + 1% glucose (**Panels e,f**: Nitrogen-Starved). Images of stem hyphae (≥ 10 µm diameter) were taken by confocal microscopy immediately after mounting the gossamers on a slide. Strain: eGFP-ATG8 at native locus. Left panels (**a,c,e**): DIC. Right panels (**b,d,f**): eGFP fluorescence. **Panels g,h**: Western blot analysis of crude protein extracts from gossamers treated as described above. The ratio free eGFP/eGFP-ATG8 bands are: 0.78 for control (C-Replete and N-Replete); 1.23 for C-Starved; and 1.23 for N-Starved. Protein load/lane = 1.25 µg. Scale bar applies to all images. Autofluorescence of the untagged strain is negligible.
