# Supplementary material for: Morphodynamics of non-canonical autophagic structures in Neurospora crassa
Source: mSphere. 2023 Oct 17;8(6):e00460-23. doi: 10.1128/msphere.00460-23 (PMC10732065; doi:10.1128/msphere.00460-23)
Supplement: Fig. S2 — Hyphae ortogonal sections. [file msphere.00460-23-s0002.pdf]

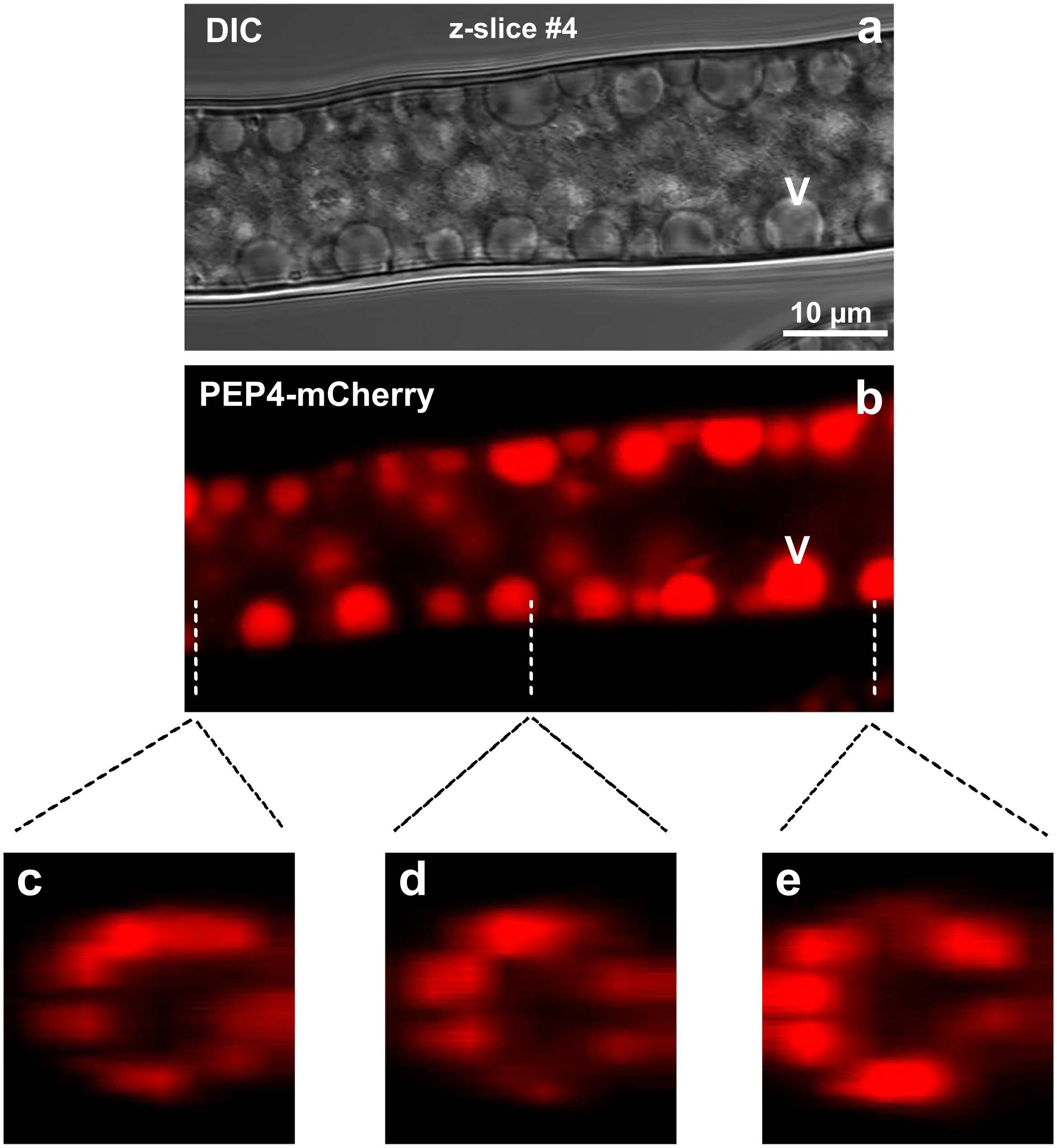

Supplemental Figure 2. ***De novo vacuoles distribute close to the plasma membrane and arrange as a sleeve along the hypha.*** **Panels a**, **b**: DIC and PEP4-mCherry vacuolar protease confocal images of slice #4 of eight total z-slices acquired from a stem hypha carbon-starved for 2h. Space between slices is 3.46 μm. **Panels c**, **d** and **e** are three orthogonal views calculated at the white dashed lines (ImageJ software).
