# Supplementary material for: Morphodynamics of non-canonical autophagic structures in Neurospora crassa
Source: mSphere. 2023 Oct 17;8(6):e00460-23. doi: 10.1128/msphere.00460-23 (PMC10732065; doi:10.1128/msphere.00460-23)
Supplement: Fig. S3 — Phagophore projections. [file msphere.00460-23-s0003.pdf]

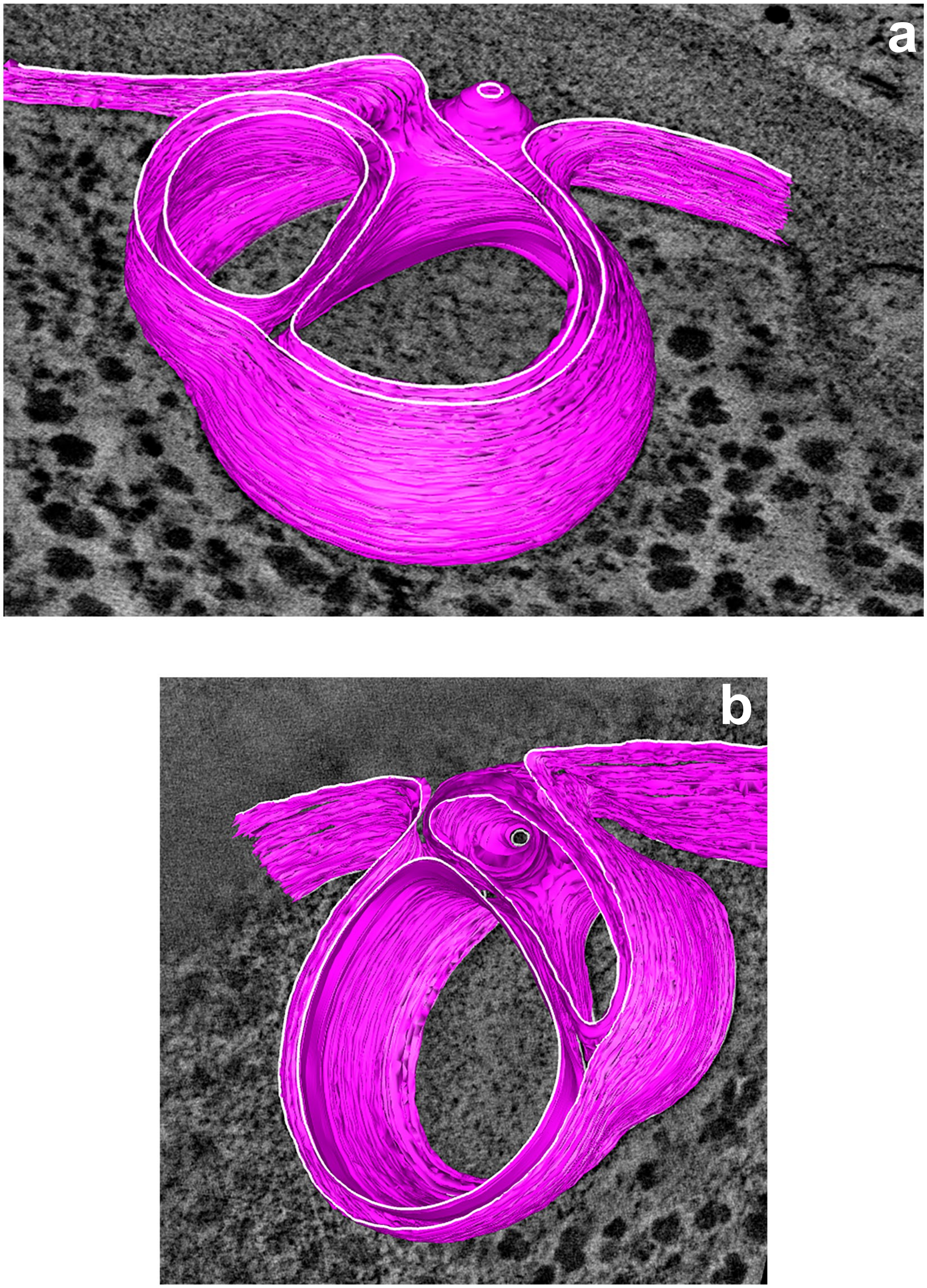

Supplemental Figure 3. ***Opposite 3D projections of the phagophore shown in Figure 14****,* to appreciate the complete structure of the complex arrangement of membranes. **Panel a**: *bottom* view, white contours drawn on membranes seen in slice view #8, slice view #184 as a background. **Panel b**: *top* view, white contours drawn on membranes seen in slice view #184, slice view #8 as a background. Dark spots outside the phagophore lumen are clusters of ribosomes.
